# The mechanisms underlying conditioning of phantom percepts differ between those with hallucinations and synesthesia

**DOI:** 10.1038/s41598-024-53663-3

**Published:** 2024-03-07

**Authors:** Magdalena del Rio, Eren Kafadar, Victoria Fisher, Rhys D’Costa, Albert Powers, Jamie Ward

**Affiliations:** 1 School of Psychology and Sussex Neuroscience, University of Sussex, Brighton, UK; 2 Yale University School of Medicine, Yale University, Connecticut, USA

**Keywords:** Human behaviour, Perception, Cognitive neuroscience, Computational neuroscience, Learning and memory, Sensory processing

## Abstract

There are many different kinds of ‘phantom’ percepts but it is unknown whether they are united by common mechanisms. For example, synaesthesia (e.g., numbers evoking colour) and hallucinations appear conceptually and phenomenologically similar: both result in a percept that does not have an environmental correlate. Here, people with synaesthesia (n = 66) performed a conditioned hallucinations paradigm known to be sensitive to hallucination susceptibility, and we asked whether synaesthetes would show the same behavioural profile as hallucinators in this task. Repeated pairing of checkerboards with tones, and gratings with colours encourages the participant to draw on prior knowledge when asked to report on the presence of the difficult-to-detect target stimulus. Synaesthetes show increased modelled expectancies for the stimulus association across the board, resulting in a higher number of detections at all stimulus intensities. This is in contrast to the pattern observed in hallucinators, who weigh their prior beliefs more strongly than controls, giving rise to more conditioned hallucinations. Results indicate that fundamentally different perceptual processes may be at the core of these seemingly similar experiences.

## Introduction

Our experience of the world is not passively received from the senses; instead, bottom-up sensory evidence is integrated with top-down expectations to construct our percepts. The framework of Bayesian perceptual inference posits that we are constantly updating a model of the world using new information, weighted by its reliability, with the ultimate goal of minimizing prediction errors (e.g.,^1^) This iterative process is thought to take place in a hierarchical manner: higher cortical levels attempt to predict the activity at the level below and lower levels feed back any discrepancy between predicted and actual signals (i.e., a prediction error). When the prediction error has a high precision and is thereby given a higher weight, it will drive a belief update. Conversely, when the prior belief is more precise than the sensory input, the prediction error will be ignored, and the current belief maintained. If the balance is sufficiently biased towards the prior, the resulting experiences may thus even be entirely divorced from objective causes out in the world, such as in hallucinatory experiences^2^.

However, there is a rich range of phenomenological experiences on the continuum between the two extremes of putatively veridical perception and hallucinations. Among these, illusions, imagery, and synaesthesia have been construed as belonging to a unitary yet heterogeneous class of phenomena^3,4^, sometimes referred to using the umbrella term of *phantom perception*^5^. The defining common characteristic of phantom perception, namely perception in the absence of an environmental correlate, has been attributed to imbalances in top-down/bottom-up integration, yet there are also some potentially important presenting differences between distinct types of phantom perception, which must have distinct underpinnings. It thus remains unclear to what extent these different experiences share a common mechanistic basis and how they diverge.

In developmental synaesthetes, sensory stimulation in one modality (termed the ‘inducer’) reliably gives rise to additional atypical experiences (termed the ‘concurrent’). For example, someone might always experience the letter ‘A’ as red. As such, there is no ‘objective’ physical cause (i.e., light of the specific wavelength for red), thereby resembling the experience of a classic and spontaneous hallucination. Both hallucinations and synaesthesia can have more or less of a perceptual character; so-called ‘projector’ synaesthetes experience the concurrent as being externally localised in space (i.e., akin to perceiving) while ‘associators’ experience it internally^6^. Similarly the phenomenology of audio-verbal hallucinations can range from being comparable to external speech to ‘thought-like’^7^). However, unlike in a hallucination, in synaesthesia there is a corresponding and consistent mapping between inducer and concurrent. These mappings emerge early in life and may draw on learned associations (e.g., childhood toys^8^ or other regularities in the environment, such as pitch-brightness^9^). Nonetheless, adult synaesthetes can acquire novel induced experiences in experimental settings (e.g., colours for unfamiliar scripts^10^) and synaesthetes experience unusually long perceptual filling-in for newly learned colour-orientation pairings in the McCollough effect^11^. At first glance, synaesthesia therefore appears more directly related to the processes underlying associative learning and perceptual inference. These nuances point to potential differences in aetiology between different types of phantom perception, yet synaesthesia is ill-defined in terms of formal computational mechanisms.

Here, synaesthetes and controls completed an auditory-visual (AV) task designed to induce conditioned hallucinations by learning to associate a checkerboard stimulus with an auditory tone^12,13^. As the experiment progresses, the predictive strength of the association changes gradually, causing participants to report tones in the presence of a visual stimulus alone, which is termed a conditioned hallucination. Activity in auditory cortical regions responsive to the target stimulus was previously found to be higher during conditioned hallucinations, indicating that this same task is capable of inducing percepts^13^. Because the synaesthetes in our sample predominantly have colour experiences elicited by letters and numbers (grapheme-colour synaesthesia), we conducted a novel visual-visual (VV) version of the task to imitate their real-life phenomenological experience and contrasted it against the original AV version. As in past work^12–14^, participants’ underlying learning and inference process was modelled computationally using a Hierarchical Gaussian Filter (HGF^15,16^). This allowed for the estimation of latent states driving behaviour on the task in order to determine if groups perform differently because of different underlying processes.

Our initial hypothesis was that synaesthetes would show a similar pattern to that seen in hallucinators^13^ and those prone to hallucinations^17^, i.e., increased conditioned hallucinations and stronger weighting of priors over sensory evidence at the level of perceptual decision-making. Contrary to our expectations, we found that synaesthetes instead have stronger and more persistent beliefs in the predictive strength of the conditioned stimulus, stemming from differences in belief-updating and leading to more stimulus detection reports overall.

## Results

**Figure 1:**
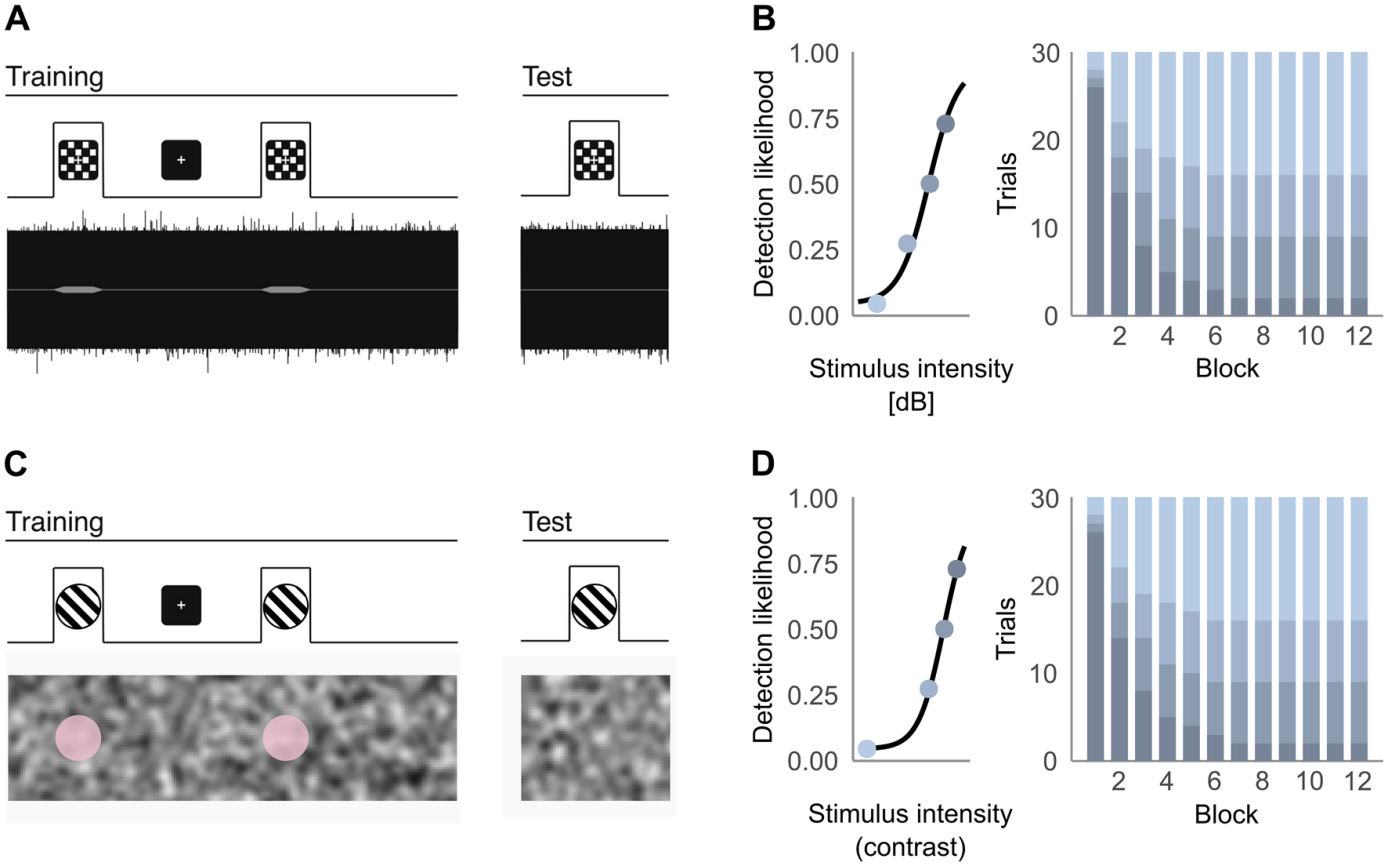
Example trial in (**A**) the auditory-visual association task and (**C**) the novel visual-visual association task. Individual psychometric curves determined the stimulus intensity presented in each condition (0%, 25%, 50% and 75% detection likelihood)—detection likelihood decreases over the 12 blocks of the experiment, as seen in the proportion of trials from each condition by block (**B** and **D**).

In two online tasks, participants were simultaneously presented with a cue stimulus (a checkerboard or a grating) and a target stimulus (a tone or a pink hue), thus establishing an association between the two in the training phase. In the subsequent testing phase, the contingency of the cue and the target was progressively reduced, such that increasingly more trials contained the cue stimulus alone, eliciting yes responses in target-absent trials or ‘conditioned hallucinations’ (see Fig. 1).Detection rates, and particularly conditioned hallucinations (yes responses in the absence of the target), constituted the key behavioural metrics of interest, as higher rates suggest a higher weight of prior knowledge relative to the immediate sensory input. This weighting ratio was additionally estimated using a computational model of the underlying inference processes, which also allows for the comparison of participants’ estimated beliefs over time.

### Behavioural results

**Figure 2:**
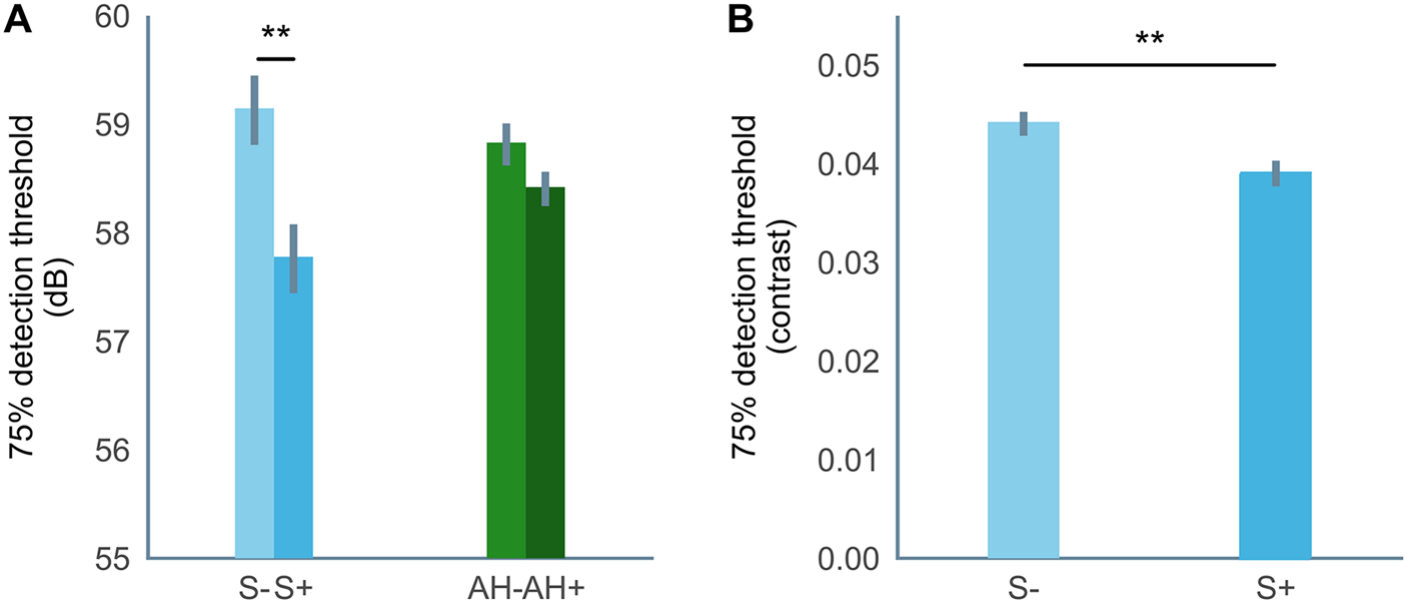
75% detection thresholds per group in (**A)** the auditory-visual task and (**B)** the visual-visual task. Summary data for the sample of individuals with auditory hallucinations and the respective control sample reported previously^12^ is included for the auditory-visual task for comparison. Error bars depict the standard error of the mean. *p < 0.05, ** p < 0.01, *** p < 0.001.

Prior to the tasks, synaesthetes had significantly lower 75%-likelihood detection thresholds compared to controls in both modalities (auditory: t(152.01) = − 3.039, p = 0.003; visual: t(149.37) = − 2.832, p = 0.005; see Fig. 2). Given that synaesthetes were presented with weaker stimuli in the tasks, we further explored the robustness of our findings using ‘matched pairs’ of participants equated for their initial detection threshold to isolate the effects of the conditioning (see Methods).

**Figure 3:**
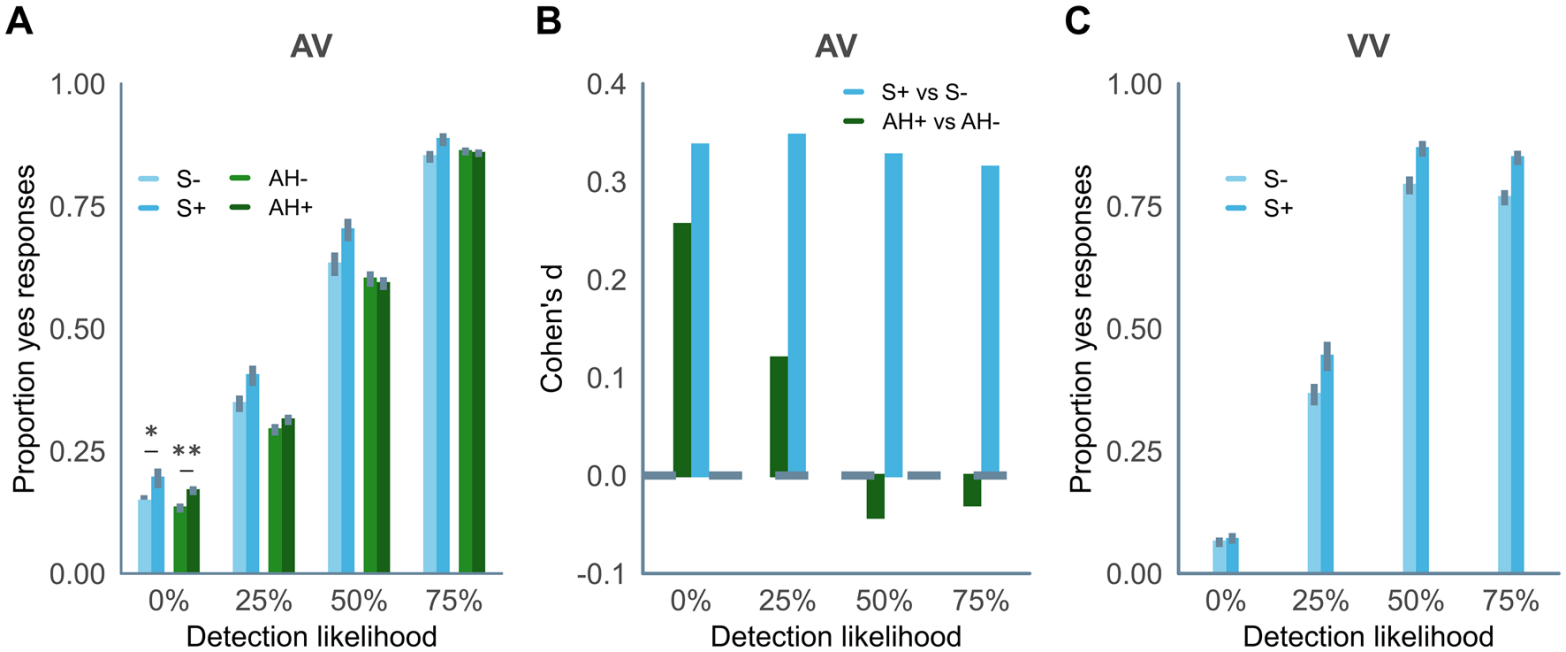
Proportion of yes responses per group in (**A**) the auditory-visual task and (**C**) the visual-visual task. A repeated measures ANOVA shows a significant main effect of group on the proportion of yes responses, such that synesthetes had more yes responses than controls in both the auditory-visual and the visual-visual tasks. Summary data for the sample of individuals with auditory hallucinations and the respective control sample who completed the auditory-visual task reported previously^12^ is included for comparison. The effect sizes in (**B**) are obtained by comparing the proportion of yes responses in the auditory-visual task in synaesthetes and individuals with auditory hallucinations to the respective control samples. Error bars depict the standard error of the mean. * p < 0.05, ** p < 0.01, *** p < 0.001.

In the main task, synaesthetes had more yes responses than controls in both tasks (AV: F(1, 156) = 7.717, p = 0.006; VV: F(1,153) = 13.769, p < 0.001). However, the pattern of results differs between the AV and VV tasks as well as between synaesthetes and those prone to hallucinations (data previously reported^12^). Synaesthetes tended to report more auditory conditioned hallucinations than controls (t(115.63) = 1.999, p = 0.048 (insensitive BF = 1.501, [− Inf, 0.537]). More importantly, synaesthetes’ increased tendency to respond yes relative to controls was present to a very similar degree at all stimulus intensities in the AV task (all Cohen’s d ranging between 0.31 and 0.35), i.e., it was not specific to conditioned hallucinations in the no-target condition (see Fig. 3A and B). In contrast, for the group with auditory hallucinations, the increase in yes responses scaled inversely with stimulus intensity, such that they had more yes responses than controls during no-tone trials, but not at any other intensity (see Fig. 3B). In the VV task, synaesthetes had more yes responses than controls at all stimulus intensities *except* in the no-target condition (t(147.39) = 0.545, p = 0.587, sensitive null BF = 0.163 [0.047, 9.733 × 10^153^], see Fig. 3C). In line with this pattern of results, synaesthetes’ objective performance as indexed by the signal detection measure d’ was significantly higher than controls’ in the VV task (t(148.36) = 2.572, p = 0.011), but not the AV task (t(145.67) = 0.440, p = 0.661; see Supplementary Fig. 9A and B). While the signal detection measure of criterion tended to be lower in synaesthetes compared to controls in the VV task, the differences were not significant in either the AV task (t(125.76) = − 0.532, p = 0.596) or the VV task (t(142.22) = − 1.787, p = 0.076; see Supplementary Fig. 9C and D). Results did not differ when participants were matched on the initial threshold (see Supplementary Materials).

To summarise the behavioural results, synaesthetes have better perceptual abilities than controls (in terms of initial thresholds, and in terms of colour detection in the main task) which was not observed in those with hallucinations, as reported previously^12^. Additionally, synaesthetes differed from those prone to hallucinations, in that they did not display increased condition hallucination rates relative to controls in the VV task and not convincingly in the AV task, as more yes responses relative to controls were found across all stimulus intensities.

### HGF results

**Figure 4:**
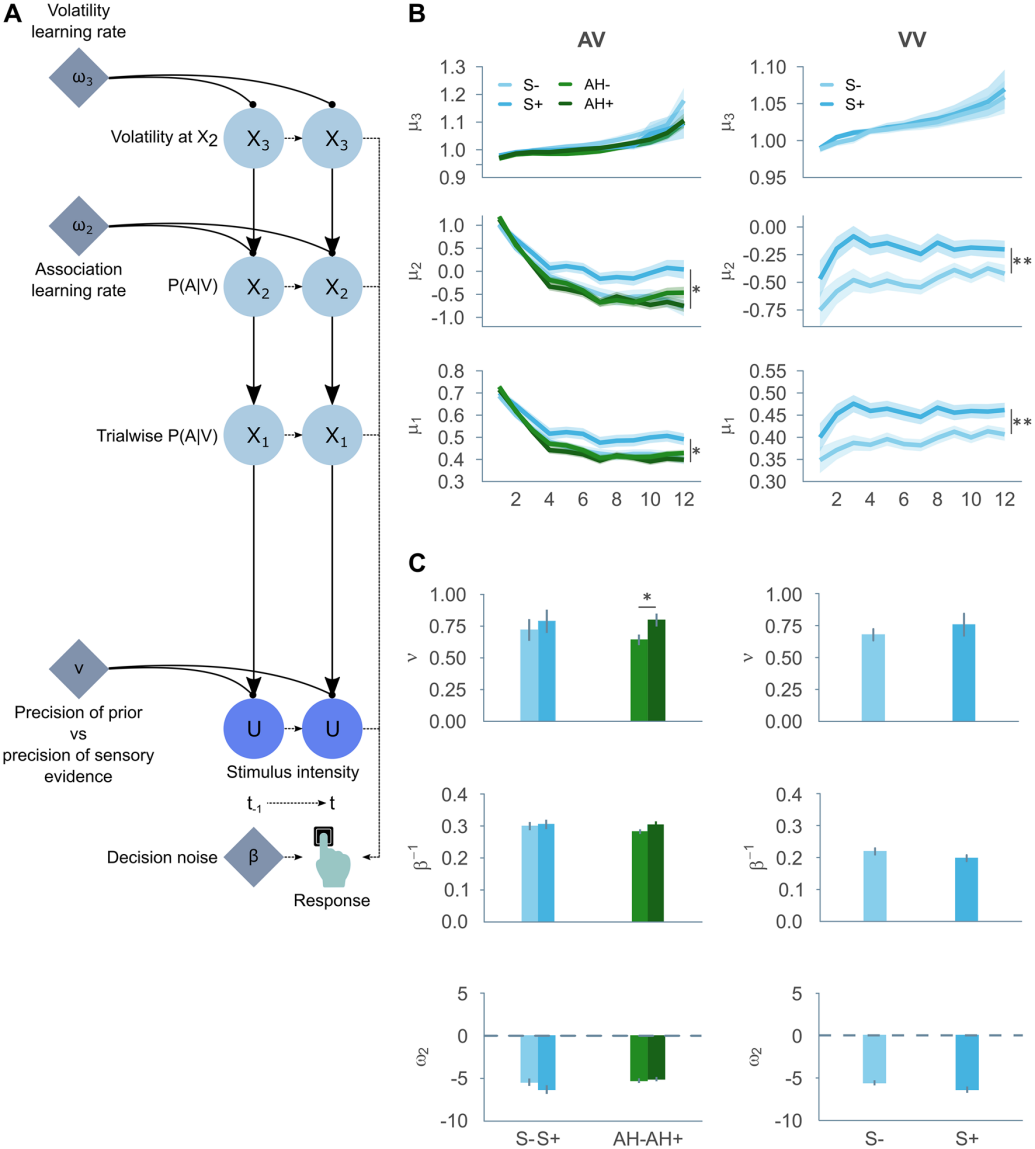
HGF analysis: (**A**) Schematic of the three-level HGF model, used to derive the belief trajectories (**B**) and parameters (**C**) in the auditory-visual and visual-visual tasks. (**B**) Estimated belief trajectories per group: X_1_ represents the belief that the conditioned stimulus was present or not on a given trial, X_2_ represents the belief that the cue and the conditioned stimulus are associated, and X_3_ represents the belief in the volatility of the association, where μ_1-3_ is the current belief or posterior at the corresponding level X_1-3_. Shaded regions depict the standard error of the mean, asterisks refer to the main effect of synaesthesia. (**C**) Estimated parameters ν (indexing the relative weight of the prior compared to the sensory evidence), β^−1^ (indexing stochasticity of response), and ω_2_ (indexing the estimate of baseline environmental volatility) per group. Error bars depict the standard error of the mean. Summary data for the sample of individuals with auditory hallucinations and the respective control sample reported previously^12^ is included for the auditory-visual task for comparison. * p < 0.05, ** p < 0.01, *** p < 0.001.

To evaluate the underlying perceptual inference process, participants’ behavioural responses were fit to a three-tiered HGF^15,16^. This model allows for the estimation of key parameters driving the rate of conditioned hallucinations, as identified in previous work^12,13^, as well as participants’ belief trajectories over the course of the paradigm. (see Fig. 4A). Here, the first level (X_1_) represents the belief that the conditioned stimulus was present or not on a given trial, the second level (X_2_) represents the belief that the cue and the conditioned stimulus are associated, and the third level (X_3_) represents the belief in the volatility of the association (for further details on the model, see Supplementary Materials).

Synaesthetes had consistently stronger beliefs than controls in the target being present and in the association of cue and target in both the AV (X_1_: F(1,156) = 4.697, p = 0.032; group × block interaction: F(5.69, 888.18) = 1.806, p = 0.099; X_2_: F(1,156) = 6.318, p = 0.013; group × block interaction: F(4.18, 652.29) = 3.157, p = 0.013; X_3_: F(1,156) = 0.771, p = 0.381; group × block interaction: F(1.25,194.53) = 1.081, p = 0.315; see Fig. 4B) and the VV task (X_1_: F(1,153) = 10.265, p = 0.002; group × block interaction: F(3.19, 487.55) = 0.754, p = 0.528; X_2_: F(1,153) = 9.576, p = 0.002; group × block interaction: F(3.16, 484.16) = 0.671, p = 0.578; X_3_: F(1,153) = 0.173, p = 0.678; F(1.21, 184.76) = 0.081, p = 0.823; see Fig. 4B). This manifested differently depending on the task, whereby it should be noted that the average belief trajectories point to inherent task differences: beliefs in the target presence and its association with the cue tended to decay over time in the AV task whereas in the VV task they increased or remained relatively constant. That said, in the AV task the starting point for beliefs in the target presence and its association with the cue was similar for both groups, yet synaesthetes’ beliefs decayed more slowly than controls’ over the course of the experiment. In the VV task, synaesthetes began the task with increased beliefs in the target presence and its association with the cue relative to controls (though note that this difference in starting point was driven by the discovery sample, see Supplementary Fig. 3).

There is no convincing evidence that synaesthetes weight these priors more strongly relative to sensory evidence than controls, as quantified by the parameter ν in either task (AV: t(137.84) = 0.730, p = 0.466, insensitive BF = 0.887 [− Inf, − 0.504]; VV: t(131.77) = − 0.174, p = 0.862, insensitive BF = 0.734 [− Inf − 0.295]; see Fig. 4C). The post-perceptual parameter β^−1^, that indexes stochasticity of response (inverse decision temperature) was likewise not significantly different for synaesthetes and controls in either task (AV: t(143.5) = 0.276, p = 0.783, sensitive null BF = 0.124 [0.055, 9.481 × 10^153^]; VV: t(152.78) = − 1.524, p = 0.130, sensitive null BF = 0.269 [0.126, 9.481 × 10^153^]). Finally, parameter ω_2_, which is an estimate of baseline environmental volatility and thus a tendency to attend to new information, while lower in synaesthetes, was not significantly different from controls’ (AV: t(140.64) = − 1.301, p = 0.195, insensitive BF = 0.577, [− Inf, − 4.613]; VV: t(140.09) = − 1.689, p = 0.094, insensitive BF = 0.754 [− Inf, − 5.888]).

In sum, synaesthetes showed more liberal detection responses than controls, reporting more stimuli in the auditory modality at all stimulus intensities and in the visual modality only when a stimulus was presented. Computational modelling suggests that this is not attributable to an overweighting of prior beliefs relative to sensory evidence as in those with hallucinations^12,13^. Instead, the model indicates that synaesthetes have and maintain higher expectancies than controls of the target being present and associated with the cue.

### Exploratory analyses: individual differences amongst synaesthetes

These are reported in full in the Supplementary Materials. Our two proxy measures of the strength of synaesthesia—number of synaesthesia types and localisation score (external projection of colours)—did not significantly correlate with the main variables of interest (the number of conditioned hallucinations, and parameters ν, β^−1^, and ω_2_) in either the AV or VV task. Nonetheless, given the results for synaesthesia overall, it may be expected that any individual differences within synaesthesia would be found in the belief trajectories. The findings were indeed suggestive of a potential effect of the localisation score in particular (i.e., being a projector). Synaesthetes who project colours externally had significantly stronger volatility expectancies, which increased more readily in both tasks, and updated their beliefs in the target and its association with the cue significantly more slowly in the AV task, relative to synaesthetes who do not project colours externally (AV: X_1_: F(20,36) = 0.900, p = 0.589, F(98.36, 177.05) = 1.421, p = 0.022; X_2_: F(20,36) = 1.209, p = 0.302, group × block interaction: F(92.01, 165.63) = 2.840 < 0.001; X_3_: F(20, 36) = 8.362, p < 0.001; group × block interaction: F(22.52, 40.54) = 7.165, p < 0.001; VV: X_1_: F(22, 34) = 0.556, p = 0.925; X_2_: F(22, 34) = 0.547, p = 0.930, X_3_: F(22, 34) = 10.955, p < 0.001; group × block interaction: F(23.18, 35.82) = 8.468, p < 0.001). The number of types of synaesthesia did not modulate the belief trajectories in the AV task, while in the VV task the volatility estimate and its learning rate were lower in synaesthetes with more types relative to synaesthetes with fewer types (AV: X_1_: F(8, 57) = 0.566, p = 0.801; X_2_: F(8, 57) = 0.640, p = 0.741; X_3_: F(8, 57) = 0.698, p = 0.691); VV: X_1_: F(9, 60) = 0.463, p = 0.894, X_2_: F(9, 60) = 0.479, p = 0.883, X_3_: F(9, 60) = 2.348, p = 0.024; group × block interaction: F(9.19, 61.26) = 4.264, p < 0.001). That is, individuals with more intense synaesthesia do not simply have a more extreme profile but rather show other differences compared to individuals with less intense synaesthesia. Dividing the synaesthetes in terms of the presence/absence of auditory inducers (e.g., sound-colour synaesthesia) or auditory concurrents (e.g., hearing motion) did not reveal significant differences (although the statistical power to detect these is reduced). In general, the results do not appear to be driven by one particular subset within our synaesthesia sample (see Supplementary Materials).

## Discussion

Different forms of phantom perception (i.e., perceptual experiences in the absence of sensory input) are often grouped together and explained via the same mechanism of greater reliance on top-down versus bottom-up information. On this basis, we hypothesised that individuals who experience synaesthesia would show a similar pattern of results as previously reported in those with hallucinations^12,13^. Instead, our findings point to both commonalities and differences between these two populations. In terms of commonalities, our results demonstrate that synaesthetes report comparable rates of auditory conditioned hallucinations as individuals with hallucinations^12^. Here the commonalities end. In contrast to hallucinators, synaesthetes also report more detections at all other tone intensities, where stimuli are actually presented. Overall, this more liberal response pattern conforms to the differences in the underlying inference process estimated by computational modelling. Synaesthetes do not overweight their priors relative to the sensory evidence, whereas previous studies have shown individuals prone to hallucinations do. Instead, synaesthetes have stronger expectancies for the target presence (X_1_) and its association with the cue (X_2_), which become fixed, implying differences related to updating their model of the environment.

Overall, synaesthetes’ beliefs remain strong despite diminishing evidence in support of the association between the cue and the target or further task experience. Our modelling results thus dovetail with a previous predictive processing account of synaesthesia. Seth^18^ has attributed the resistance of synaesthetic associations to prediction errors (encountering inducers in the absence of the concurrent) to the encoding of synaesthetic associations in intermediate levels with unusually high precision. According to this proposal, higher-level models would be reshaped over time to accommodate the synaesthetic percepts. The most parsimonious explanation of the results in both tasks may indeed be that intermediate-level beliefs become fixed, whereby the specific implementation mechanism for this remains unclear, as possibilities are varied. The possibilities include the learning rate ω_2_, which reflects the attention paid to new information, aberrant precision weights on the prediction error and aberrant precision-weighted prediction error (parameters ψ and ε, respectively). Exploratory analyses of these parameters did not reveal significant group differences (see Supplementary Fig. 22) but nor was the current study optimized to do so. Pinpointing the exact mechanism underlying atypical belief-updating in synaesthesia may benefit from testing a paradigm featuring high- and low-volatility conditions, as opposed to the gradual drift in association contingencies used here. In fact, there is evidence of synaesthetes’ learning local statistics at the same rate as controls yet requiring greater evidence accumulation to discern global statistics in a pseudorandom game of whack-a-mole ^19^. It would be informative to replicate these findings in conditions that emulate synaesthesia, such as our conditioned hallucinations paradigm. Alternative computational models, including self-reinforcing expectancies (e.g.,^20^) or a circular inference framework^21^ may also aid in the characterisation of the underlying belief updating processes.

Irrespective of the precise algorithmic implementation, the current paradigm revealed differences in belief updating evocative of the theory of synaesthesia according to which certain stimulus pairings learnt in childhood are never forgotten (e.g.,^22^). While idiosyncratic synaesthetic mappings are prevalent, they are on average not random, but rather often attributable to environment statistics, such as the direct exposure to letter-colour pairings, letter frequency, similarities between letters, and semantic factors such as the first letter of colour terms^8,23,24^. Beyond grapheme-colour synaesthesia, it is common to find cross-modal correspondences across sensory features such as sour being sharp, and high pitch being bright in synaesthetic associations^9^. Atypical learning after exposure to certain environment statistics may thus be one contributing factor to developmental synaesthesia, yet it does not constitute a full account. Indeed, the VV task does not induce conditioned hallucinations to the same degree as graphemes do in synaesthetes’ daily lives. There are two characteristics of synaesthesia that are not captured in the current paradigm and may provide further insights. One is the fact that synaesthetes do not tend to experience isolated pairings, but rather multiple unique associations between the elements of two given systems, a process termed by some researchers veridical mapping^25^. A second characteristic is that the concurrent is generally task-irrelevant in synaesthesia. In the current study, the conditioned stimulus is the target, yet if a synaesthete is presumably learning to read, their goal will be to identify letters or words, not the colour in which they are printed. A spurious correlation with a task-irrelevant feature may not be advantageous, but neither will it necessarily be disadvantageous. Emulating these distinctive aspects of the phenomenon may further our understanding of its development in future research.

This type of data may be required to fully elucidate the role of the sensory modalities involved in phantom experiences. While we do not have data testing individuals with hallucinations on the VV paradigm, and are thus unable to test for an interaction between modality and group, there is evidence for both modality-specific and modality-general effects in those with hallucinations, e.g., the hallucination-prone tend to overweigh priors also in a visual task^26^. Similarly, conclusively teasing apart modality-specific effects congruent with the synaesthesia modality would require a more selective recruitment process that enabled a direct comparison of synaesthetes with exclusively visual concurrents and synaesthetes with exclusively auditory concurrents. That said, synaesthetes’ characteristic profile was broadly consistent across both AV and the novel VV task developed to mimic grapheme-colour synaesthesia, whereby there were two notable differences across tasks. At the behavioural level, synaesthetes had similarly enhanced detection rates at all stimulus intensities in the AV task, whereas detection rates were rather increased at all stimulus intensities except during the no-hue condition in the VV task, resulting in significantly improved performance as quantified by the signal detection theory measure d’ in this task. At the level of the estimated belief trajectories, synaesthetes’ expectancies extinguished more gradually in the AV task, and had stronger initial priors or learning rates in the VV task. Given that our sample consisted predominantly of synaesthetes with visual experiences, a perceptual advantage in the visual modality would be in line with prior research^27^. However, synaesthetes also tended to have objectively better performance on the AV task and had lower detection thresholds prior to conditioning in both the auditory and visual modalities. Indeed, a recent study has showed a larger EEG-based N1 auditory evoked potential to simple sounds in synaesthesia^28^. It is therefore possible that our pattern of results reflects genuine differences in perceptual sensitivity, although note that differences in d’ are only significant in the VV task. Likewise, differences in belief trajectories across tasks may be attributable to modality-specific effects, yet additional evidence from the exploratory individual differences analysis was inconclusive. Importantly, it should be noted that discrepancies across AV and VV associations may also arise due to differences between the tasks themselves. Across both groups, conditioned hallucinations are less frequent in the VV task and both objective performance and beliefs across blocks increases rather than decreases asymptotically, suggesting a process more akin to perceptual learning in this task than in the AV task (see Fig. 4 and Supplementary Fig. 10).

Another aspect that may be thought to affect the mechanistic underpinnings of synaesthesia is its subjective character. Previous research using dynamic causal modelling indeed points to a bottom-up pathway in projector synaesthetes and a top-down pathway in associators^29^. However, our exploratory analysis shows primarily quantitative variation along the projector-associator spectrum. Projectors do not have more conditioned hallucinations or overweight their priors relative to sensory evidence more than associators (see Supplementary Fig. 18). Instead, they have particularly strong and fixed expectancies for the auditory target and its association with the cue, and stronger volatility expectancies (see Supplementary Fig. 17). Given that learning rates should increase in high-volatility environments, this suggests a more pronounced insulation of belief updating from changes in the environment statistics.

Similarly, one should consider the perceptual nature of the conditioned hallucinations elicited in the task. Here, we do not ask the participant to self-report on this, and thus cannot definitively know to what extent these experiences had a genuinely vivid perceptual character across all participants and trials. However, previous work shows several features of conditioned hallucinations which support their perceptual character. Individuals with hallucinations report more targets exclusively in trials with sparse incoming evidence, and with higher confidence than controls^12,13^, which does not fit the profile of a simple response bias. In addition, neural activity during conditioned hallucinations resembles that observed in studies of clinical symptom capture during hallucinations in the MRI scanner^13^. In conclusion, we find unique behavioural profiles for different types of phantom perception that map onto different computational estimates, allowing us to discriminate between populations in mechanistic terms within the same task and model. While synaesthetes appear to have some traits in common with hallucinators, they do not overweight their priors relative to sensory evidence but rather show differences in their belief-updating resulting in stronger expectancies and liberal response patterns. This suggests that not only does ‘non-veridical’ perception manifest in the cognitive processes tapped into by this task, but also that the type and quality of these experiences may provide meaningful clues regarding the underlying mechanisms of unusual perceptual experiences.

## Methods

Two samples of synaesthetes and controls were collected. The methods and analyses for the replication sample were pre-registered based on the findings of the discovery sample (10.17605/OSF.IO/BCGS4). Specifically, the discovery sample was used to develop the exclusion criteria for the novel visual-visual task and as the basis for a power analysis. However, the task procedure from the participant’s perspective was identical and the two samples are combined in the main report for ease of exposition. Any differences between samples are noted in the text and reported in full in the Supplementary Materials (e.g., similar trends reaching significance in one sample but not the other). The data on individuals with synaesthesia collected as part of the present study are compared to summary data on individuals with auditory hallucinations reported previously^12^.

### Participants

Ninety-six synaesthetes (78 female, 11 male, 7 undeclared, M_age_ = 29.5, SD_age_ = 9.9), and 117 non-synaesthetes (88 female, 26 male, 3 undeclared, M_age_ = 31.7, SD_age_ = 9.9) participated in the study. After exclusion criteria were applied (see Supplementary Materials), 66 synaesthetes (56 female, 8 male, 2 undeclared, M_age_ = 30.5, SD_age_ = 9.6) and 92 controls (74 female, 16 male, 2 undeclared, M_age_ = 31.4, SD_age_ = 8.9) remained for the AV task and 71 synaesthetes (58 female, 7 male, 6 undisclosed, M_age_ = 30.6, SD_age_ = 10.3) and 84 controls (71 female, 12 male, 1 undeclared, M_age_ = 31.6, SD_age_ = 9.4) remained for the VV task.

All synaesthetes were recruited via e-mail from the University of Sussex synaesthesia database. They minimally all have synaesthetic colour experiences and have been verified with test–retest consistency. Most were verified for grapheme-colour consistency (a score of less than 1.43 as used previously^30^), two took an equivalent test of sound-colour, and one was verified for sequence-space synaesthesia as they had few colours (method reported previously^31^). Additional types of synaesthesia varied across subjects (see Supplementary Materials). The control group in the discovery sample primarily consisted of students at the University of Sussex and received either course credits or cash as compensation for taking part in the experiment. The control group in the replication sample were recruited using Prolific, and thus only received compensation in cash at the same rate of £9/hour as the synaesthetes. All controls were selected to match the synaesthete sample in age range and female:male ratio. All participants reported normal or corrected-to-normal vision and hearing. Participants recruited through Prolific were additionally required to report fluency in English, a minimum of 400 previous submissions and a 90% approval rate on Prolific. The advert on Prolific clearly stated that participants should not take part if they believed they have synaesthesia, and the question was included together with general demographic questions.

The study was performed in accordance with the relevant guidelines and regulations and approved by the local ethics committee of the University of Sussex (reference number ER/RD381/1). All participants gave written informed consent.

### Materials and procedure

Participants completed two online sensory detection tasks which use associative learning to induce yes responses in target-absent trials (‘conditioned hallucinations’) in the auditory and visual modality respectively. Individual psychometric thresholding determined the intensity of the target stimuli in each modality prior to the main task. Early in the experiment, the cue and the target were presented concurrently at 75% detection likelihood intensity, fomenting an association between the two. The strength of this association was tested over the course of the experiment, during which the likelihood of 75% threshold-level target presentations decreased non-linearly, while the likelihood of trials where the target was absent or at subthreshold intensity increased. In the original AV version of the task^12,13^, participants indicated the detection of an auditory target consisting of a 1-kHz pure tone embedded in 70-dB broadband white noise, cued by a black-and-white checkerboard. In the VV version of the task developed here to emulate grapheme-colour synaesthesia, the target instead consisting of pink hue, which was cued by an annular sine-wave grating. The tasks are described in full in the Supplementary Materials, which includes further details on the steps taken to address the limitations of testing online.

### HGF analysis

Participants’ underlying inference process was modelled using a three-level HGF model (see Supplementary Materials). This is a general model of Bayesian learning based on sequential input in a volatile environment which has been previously implemented in the analysis of the AV task, whereby model parameters are updated based on participant responses and stimulus intensities^12,13^.

### Statistical tests

Detection thresholds were compared across synaesthete and control groups using Welch’s t tests. The proportion of detection reports were submitted to a two-way repeated-measures analysis of variance (ANOVA) with the factors signal intensity and group (synaesthete and control). The key comparison of detection reports in the no-stimulus condition was tested with a post-hoc Welch’s t test. Performance was additionally characterised using the signal detection measures d’ and criterion, by considering detection reports in the no-target condition as false alarms and those at all other intensities as hits. Performance evolution over the course of the experiment was also submitted to a three-way repeated measures ANOVA with the additional factor of block number. The parameters derived from the model (ν, β^−1^ and ω_2_) were compared between synaesthete and control groups using Welch’s t tests, though note that ω_2_ tests were not pre-registered. The modelled belief trajectories were compared by means of a mixed ANOVA per level (X_1_, X_2_ and X_3_). The Greenhouse–Geisser correction was applied as needed throughout.

While the synaesthete and control groups were matched on perceptual difficulty through the staircase procedure prior to the task, synaesthetes had lower perceptual thresholds than controls and were, on average, presented with quieter tones and fainter colours. To explore the impact of this, we created a matched subgroup of pairs of synaesthetes and controls based on the absolute value of their initial 75% detection likelihood thresholds using nearest-neighbour matching with a caliper of 0.1 standard deviation units based on logistic regression propensity scores and implemented with the MatchIt R package^32^. The analyses conducted on this matched subgroup can be regarded as exploratory insofar as it was not pre-registered, yet they act as a test of the robustness of the main findings against this possible confound (see Supplementary Materials).

We had pre-registered that Bayes factors would be calculated using the discovery dataset as the prior. However, because we are reporting the results of the pooled samples, we instead base these on the effect sizes reported previously comparing hallucinators and controls^12^. Priors were thus defined as a half-normal distribution with a mean at 0 and an SD of the value of the difference between individuals with hallucinations and controls divided by two. All Bayes factors are reported together with the robustness region calculated using a custom MATLAB script. All tests were performed separately for the AV and the VV task.

We had hypothesised that any differences between synaesthetes and controls would be stronger in individuals with more intense synaesthesia. Synaesthesia intensity was quantified by two proxy measures: (1) the number of types of synaesthesia^33^ and (2) the localization subscale of the Coloured Letters and Numbers questionnaire (CLaN^30^), where a high score indicates a tendency to experience synaesthetic colours as externally localised. In addition, we investigated the modality-specificity of the group differences. While all synaesthetes in our sample had visual experiences (mainly grapheme-colour synaesthesia), emulated by the VV task, only a subset additionally reported having types of synaesthesia involving audition, specifically hearing-motion synaesthesia (HMS, i.e., a type of visual-to-auditory synaesthesia^34^) and/or auditory-to-visual synaesthesia (AVS). Individual differences were assessed via Spearman’s correlations or Welch’s t tests on the key summary measures (the number of conditioned hallucinations, and parameters ν, β^−1^, and ω_2_), and via repeated measures ANCOVAs or ANOVAs on the belief trajectories.

### Supplementary Information

Supplementary Information.

## Data Availability

Relevant model code has been made freely available as part of the TAPAS computational toolbox (www.github.com/translationalneuromodeling/tapas). De-identified data and analysis scripts which do not already form part of the TAPAS computational toolbox are available on OSF: 10.17605/OSF.IO/ZTY6M.
