# Supplementary material for: The mechanisms underlying conditioning of phantom percepts differ between those with hallucinations and synesthesia
Source: Sci Rep. 2024 Mar 7;14:5607. doi: 10.1038/s41598-024-53663-3 (PMC10920618; doi:10.1038/s41598-024-53663-3)
Supplement: Supplementary file 1 — Supplementary Information. [file 41598_2024_53663_MOESM1_ESM.pdf]

Supplementary Materials

1. Conditioned hallucinations paradigm

1.1. Task design

The tasks were implemented via React (https://reactjs.org), using the same structure as previous versions[1]. In the auditory-visual (AV) task, the visual stimulus (the cue) consisted of a black-and-white checkerboard on a black background. The target auditory stimulus consisted of a pure 300-ms 1-kHz tone of varying loudness, embedded in 70-dB broadband white noise. In the visual-visual (VV) task, the cue stimulus consisted of 256 × 265 px annular sine-wave gratings with a spatial frequency of 80 px per degree embedded in visual white noise. The orientation of the grating was randomised across subjects, yet constant throughout the experiment within-subject. A low-contrast pink hue (the target) was superimposed on the gratings at varying intensities.

As the tasks were conducted online, we implemented several measures to make testing conditions comparable across a heterogeneous array of stimulus presentation set-ups. Participants were instructed to wear headphones and set both the volume and screen brightness to the maximum value. They completed two qualifying tasks to ensure they complied with these instructions. In the auditory qualifying task, participants were asked to identify the quietest of a sequence of three tones. The task was designed to be nearly impossible without the use of headphones by using wave interference phenomena[2]. In the visual qualifying task, participants were asked to identify a shape with a minimally different hue from the background, such that a high level of screen brightness was required to correctly identify the shape. A minimum of 80% accuracy was required in these tasks to proceed.

Next, individual 75% detection likelihood thresholds for the target intensity (i.e., the loudness of the target auditory stimulus in the AV task and the contrast of the pink hue in the VV task) were estimated using two interleaved 40-trial long staircases using QUEST, a maximum-likelihood based procedure adapted to JavaScript from Psychtoolbox 3.0[3,4]. Participants indicated 'yes' or 'no' using the 'q' and 'e' keys. Intensities for the 25% and 50% detection likelihood conditions were derived from a psychometric function fitted to the 75% detection likelihood data. Initial QUEST parameters were defined empirically from data acquired from several participants in person in the Yale laboratory, using various hardware configurations and systems.

Finally, participants completed the main task (AV or VV task). Each trial consisted of a fixation period lasting 500-1000 ms, followed by the concurrent presentation of the cue stimulus (a checkerboard or a grating) and the target stimulus (a tone or a pink hue). Participants reported the detection of the target stimulus through button-press after stimulus-offset within a response window

lasting 1500 ms. As in the QUEST procedure, participants used the 'q' and 'e' keys to indicate ‘yes’ or ‘no’, holding these down to indicate their degree of confidence in their responses (the longer the press, the higher the confidence in the response). This was represented on the screen with a visual analogue scale ranging from 1 'Unsure' to 5 'Certain'. Missed trials were repeated. 80% accuracy on two short practice sessions was required before the main task could begin.

During each experiment, participants incidentally learned the association between the cue and target stimuli. The strength of this association was tested over the course of 12 blocks of 30 trials, during which the non-linear decrease in 75% threshold-level target presentations was concomitant with the progressive increase in target-absent and subthreshold trials. Each block had a fixed ratio of stimulus intensities which were presented in a pseudorandomized order.

Note that after the discovery dataset for the novel VV task was collected and analysed, several measures were taken to improve the estimation of the psychometric curve: the hard-coded lower bound in the QUEST procedure was decreased from 0.01 to 0.0001 and additional limits were implemented for the 50%- and 75%-detection likelihood threshold estimates, such that the 25%-detection likelihood threshold estimate could not fall below 0. However, the task did not change from the point of view of the participant, and the key findings remained unaltered. All significant differences between samples are reported.

1.2. Exclusion criteria

Nine participants were excluded due to missing behavioural data. Two participants in the control group indicated that they may have synaesthesia and were thus excluded from the analysis. In addition, the exclusion criteria applied previously[1] were adapted to account for the novel VV task. Changes were informed by the discovery sample and preregistered for the replication. Participants were only included if

1. Fitting a linear function yielded a positive slope for the percentage of yes responses over stimulus intensity (trial type 0%, 25%, 50% and 75%),

2. Less than 10% of the reaction times were below 250 ms, and

3. The stimulus intensity estimated to result in 50% detection likelihood was higher than the stimulus intensity estimated to result in 25% detection likelihood. (This novel exclusion criterion was introduced to account for findings in the VV task, replacing the previously used criterion of the percentage of detections over the first 15 trials being over 55%).

Subsequently, we removed participants with:

1. Any outlier proportions of yes responses (defined as values that deviated from the grand mean by more than 1.5 times the interquartile range per condition)

2. Negative slope for the proportions of yes responses over stimulus intensity excluding the target absent trials (trial type 25%, 50% and 75%),

3. Slopes that deviated by more than 2 SDs from the grand mean.

1.3. HGF model

Briefly, inference on the first level of the HGF (X_1) represents the participant's belief that the target was present or not on each trial given the cue. The second level (X_2) represents the belief that the cue predicts the target, and the third level (X_3) represents the belief in the volatility of the association between cue and target, i.e., the volatility of X_2. At each timestep, the current belief μ is instantiated as the Bayesian posterior mean of a beta distribution, and updated following the equation:

$$\mu = \hat{\mu} + \frac{1}{(1 + v)} * \delta$$

i.e., based on the prior $\hat{\mu}$ as well as the divergence of the current observation from the prior or prediction error δ. Here the observation is defined as the rate of the target presented at each trial (25%, 50% or 75%), and the prior is derived by the HGF. The parameter ν determines the relative weighting of the prior vs the observation, such that if ν is large, the prior is weighted more than the observation and vice versa. Post-perceptual decision noise is modelled by a logistic sigmoid function. Specifically, parameter β^{-1} (the inverse decision temperature) modulates the slope of the sigmoid, thus capturing how deterministic the decision is, i.e., the sensitivity of the choice probability to differences in stimulus intensity values. Small β^{-1} values increase the slope and make the decision more sensitive to small differences in stimulus intensity. Parameters ω_2 and ω_3 quantify the learning rate or belief evolution rate at HGF levels 2 and 3, such that high values of ω indicate a stronger tendency to attend to new information. Note that, as this parameter is part of an exponent, this refers to the signed value of the parameter, such that an agent with ω of -4 will pay less attention to new information than an agent with ω_2 of -2.

The HGF model for the discovery dataset was fit using participants' expected target detection rates as the stimulus intensity inputs, yet there is evidence of improved model fit by instead using the empirical grand mean detection rates in heterogeneous online samples[1]. Indeed, here we replicated this finding using a Bayesian model comparison (see 6. Model Comparison). We thus performed the analysis using the empirical grand mean, which additionally allowed us to better compare findings across studies.

2. Control analyses – separate analyses for discovery and replication samples

2.1. Replication procedure

The final sample was obtained by pooling the discovery sample (33 synaesthetes and 40 controls) and the replication sample (52 synaesthetes and 64 controls). The latter sample size was decided using a power analysis based on the group differences observed in the discovery sample. Specifically, we had found that synaesthetes had a higher value for the β parameter than controls (VV: Cohen's d = 0.522, AV: Cohen's d = 0.717). A power analysis using G*Power indicated that a sample of 47 per group or samples of 58 and 38 respectively using a recruitment ratio of 1.5:1 would suffice to detect an effect of similar size as the smallest one found in our discovery sample (i.e., Cohen's d = 0.522), at the standard alpha level of 0.05 and power of 0.8, using a one-sided t test. However, note that the main findings pertain to the belief trajectories as opposed to the parameters. A post-hoc power analysis based on the smallest significant main effect size detected in the repeated measures ANOVA (X_1 in the AV task) indicated that this test achieved 72% power assuming the default correlation among repeated measures of 0.5.

2.2. Participants

The discovery sample consisted of 23 synaesthetes (19 female, 4 male, M_{age} = 28.2, SD_{age} = 9.7) and 34 controls (21 female, 13 male, M_{age} = 24.5, SD_{age} = 4.0) who completed the AV task, and 26 synaesthetes (21 female, 4 male, 1 undeclared, M_{age} = 28.8, SD_{age} = 9.9) and 27 controls (8 male, 19 female, M_{age} = 23.2, SD_{age} = 3.8) who completed the VV task.

The replication sample consisted of 43 synaesthetes (37 female, 4 male, 2 undeclared, M_{age} = 31.8, SD_{age} = 9.4) and 58 controls (53 female, 3 male, 2 undeclared, M_{age} = 35.4, SD_{age} = 8.6) who completed the AV task, and 45 synaesthetes (37 female, 3 male, 5 undeclared, M_{age} = 31.6, SD_{age} = 10.5) and 57 controls (4 male, 52 female, 1 undeclared, M_{age} = 35.6, SD_{age} = 8.6) who completed the VV task.

2.3. Behavioural results

Prior to the task, synaesthetes' lower detection thresholds were only significant for the replication sample, in the AV task (discovery sample: $t(53.75) = -0.723$, $p = 0.473$; replication sample: $t(86.691) = -3.340$, $p = 0.001$) and in the VV task (discovery sample: $t(50.78) = 1.192$, $p = 0.239$; replication sample: $t(91.781) = 2.543$, $p=0.013$; see Supplementary Figure 1).

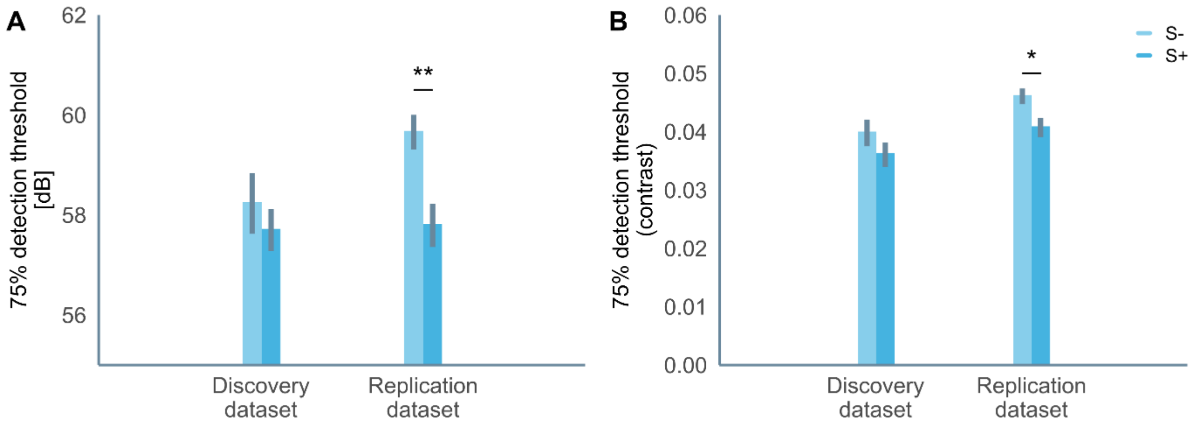

Supplementary Figure 1: Detection thresholds for synaesthetes and controls in **A** the AV task and **B** the VV task. Error bars depict the standard error of the mean. p <0.05, ** p <0.01, *** p <0.001

In the main task, synaesthetes reported detecting the target more often than controls in the replication sample in the AV task (discovery: F(1, 55) = 3.562, p = 0.064; replication: F(1, 99) = 4.356 p = 0.039) and in both discovery and replication samples in the VV (discovery: F(1, 51) = 15.531 p < 0.001; F(2.55, 130.2) = 3.136, p = 0.035; replication: F(1,100) = 4.587, p = 0.035). The crucial comparison of reported detections in the no-target condition (i.e., the number of 'conditioned hallucinations') did not differ by group for the VV task in either sample (discovery: t(44.185) = -1.075, p = 0.288; replication: t(96.674) = -0.042, p = 0.966). There was a significant difference in conditioned hallucinations in the AV task, yet this was likewise restricted to the replication sample (discovery: t(39.208) = 0.661, p = 0.513; replication: t(70.261) = -2.2463, p = 0.028).

Average d' was not significantly different in the AV task in either dataset (discovery t(47.865) = 0.739, p = 0.464; replication t(94.434) = -0.073, p = 0.942) and only significantly different in the discovery dataset in the VV task (discovery t(49.359) = 2.0582, p = 0.045; replication: t(95.739) = 1.840, p = 0.069).

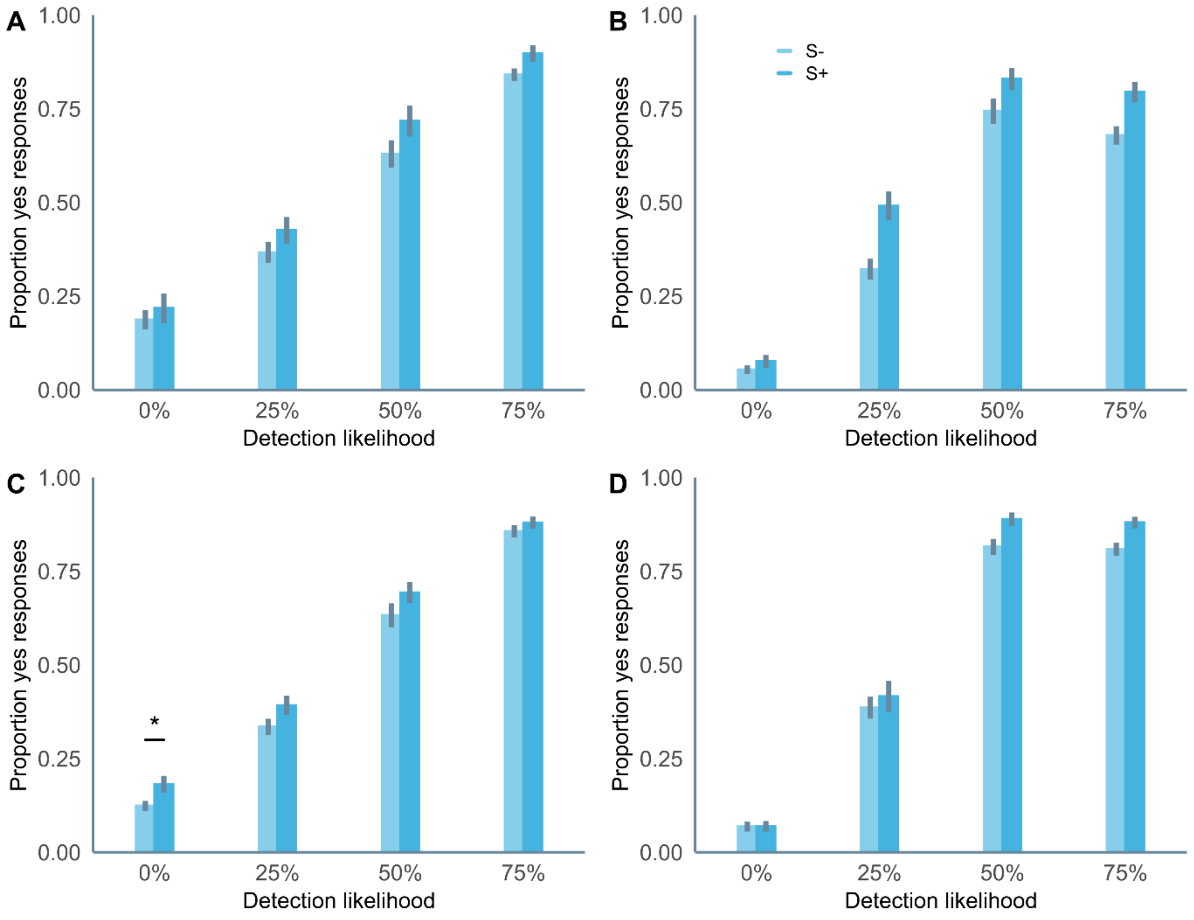

Supplementary Figure 2: Proportion of yes responses for synaesthetes and controls in the AV task for **A** the discovery sample and **C** the replication sample, and in the VV task for **B** the discovery sample and **D** the replication sample. Error bars depict the standard error of the mean. p <0.05, ** p <0.01, *** p <0.001

2.4. HGF results

Synaesthetes had consistently stronger beliefs in the target being present and in the association of cue and target in both datasets and tasks, yet this was only significant for X_2 in the replication sample in the AV task (discovery X_1: F(1, 55) = 2.243, p = 0.140; X_2: F(1, 55) = 2.475, p = 0.121; X_3 F(1, 55) = 0.490, p = 0.487; replication: X_1: F(1, 99) = 2.553 p = 0.113; X_2: F(1, 99) = 3.904, p = 0.051; group × block interaction: F(3.49, 345.87) = 3.087 p = 0.021; X_3: F(1, 99) = 0.366, p = 0.547), and for X_1 and X_2 in the discovery sample in the VV task (discovery: X_1: F(1, 51) = 13.144, p < 0.001; X_2: F(1, 51) = 12.675, p < 0. 001; X_3: F(1, 51) = 2.084, p = 0.155; replication: X_1 F(1, 100) = 2.637, p = 0.108; X_2: F(1, 100) = 2.298, p = 0.133; X_3: F(1, 100) = 0.296, p = 0.587). Note that, in addition, differences in the starting point in the VV task were driven by the discovery sample, and that significant differences in X_3 in the VV task were found in the discovery, but not the replication dataset.

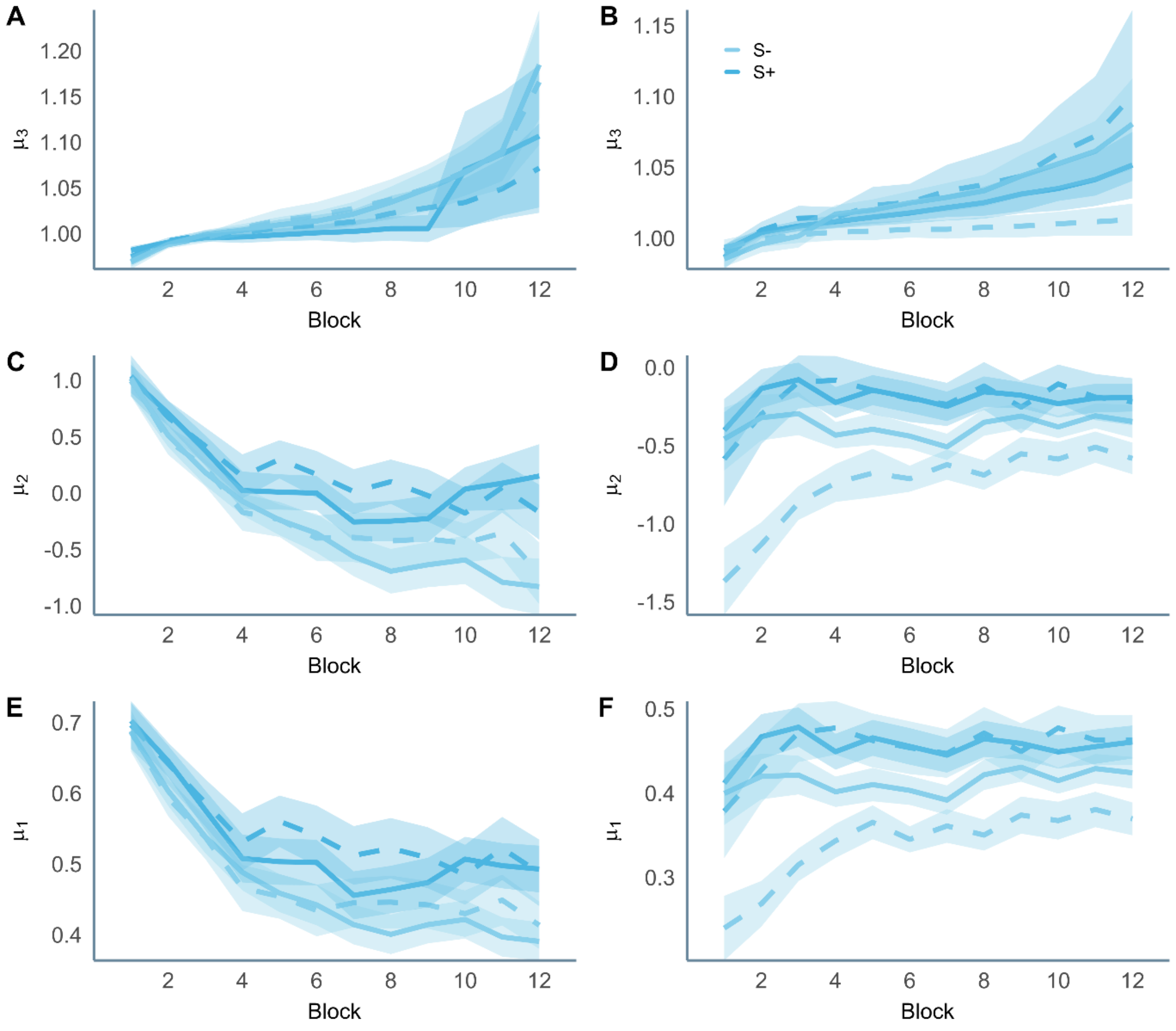

Supplementary Figure 3: Estimated belief trajectories for synaesthetes and controls per task and dataset (discovery sample in dashed lines, replication sample in continuous lines): X_1 represents the belief that the conditioned stimulus was present or not on a given trial (AV task in **E**, VV task in **F**), X_2 represents the belief that the cue and the conditioned stimulus are associated (AV task in **C**, VV task in **D**), and X_3 represents the belief in the volatility of the association (AV task in **A**, VV task in **B**) , where $\mu_{1\text{-}3}$ is the current belief or posterior at the corresponding level $X_{1\text{-}3}$. Shaded regions depict the standard error of the mean. Asterisks denoting significance are omitted for legibility.

There is no evidence that synaesthetes weight priors more strongly relative to sensory evidence than controls, as quantified by the parameter ν (AV discovery: $t(40.824) = -0.602$, $p = 0.550$; AV replication: $t(94.303) = -0.439$, $p = 0.662$; VV discovery: $t(46.442) = 0.643$, $p = 0.524$; VV replication: $t(81.322) = -0.117$, $p = 0.907$). The post-perceptual parameter β^{-1} that indexes the stochasticity of the response, or inverse decision noise, was only significantly lower in the AV task in the discovery sample (AV discovery $t(47.819) = 1.401$, $p = 0.168$; AV replication: $t(83.945) = -2.097$, $p = 0.039$; VV discovery: $t(46.483) = 0.060$, p-value = 0.952; VV replication: $t(98.394) = 1.943$, $p = 0.055$). Finally, parameter ω_2, which indexes the estimate of baseline environmental volatility and

thus a tendency to attend to new information, was not significantly different in the AV or VV task in either sample (AV discovery: $t(45.145) = 1.310$, $p = 0.197$; AV replication: $t(92.516) = 0.630$, $p = 0.530$; VV discovery: $t(45.621) = 0.437$, $p = 0.665$; VV replication: $t(91.096) = 1.735$, $p = 0.086$).

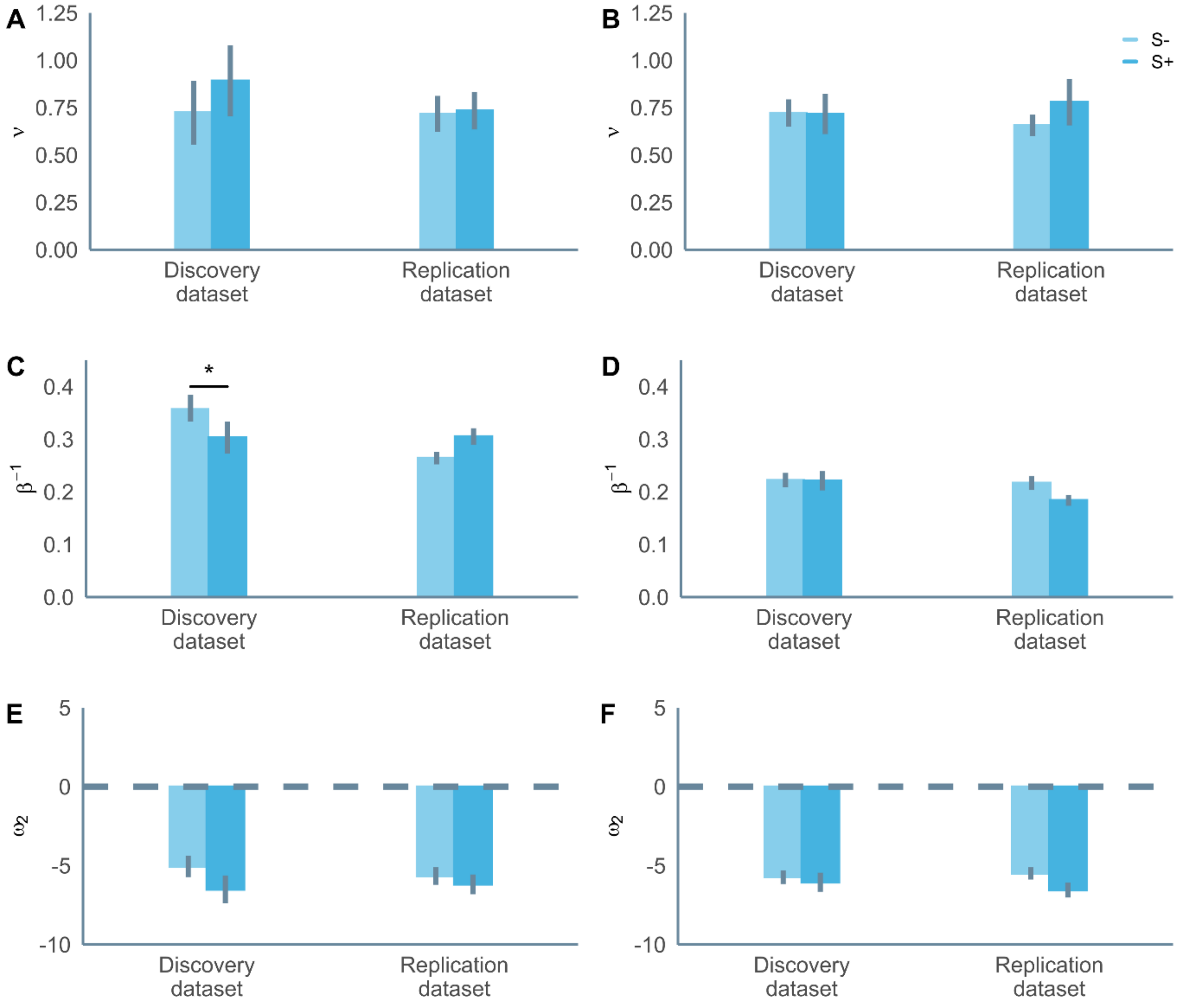

Supplementary Figure 4: Estimated parameters ν (indexing the relative weight of the prior compared to the sensory evidence; AV task in **A**, VV task in **B**), β^{-1} (indexing stochasticity of response; AV task in **C**, VV task in **D**), and ω_2 (indexing the estimate of baseline environmental volatility; AV task in **E**, VV task in **F**) for synaesthetes and controls, separately for the discovery and replication samples. Error bars depict the standard error of the mean. p <0.05, ** p <0.01, *** p <0.001

3. Control analysis - matched participants based on detection thresholds

Synaesthetes and controls were matched based on the absolute value of their initial 75% detection likelihood thresholds using nearest-neighbour matching with a caliper of 0.1 standard deviation units based on logistic regression propensity scores and implemented with the MatchIt R package [5]. In the absence of a close enough match for some of the synaesthetes, the sample size was reduced. 52 synaesthetes (42 female, 8 male, 2 undeclared, $M_{age} = 30.0$, $SD_{age} = 9.5$) and 52 controls (45 female, 6

male, 1 undeclared, M_{age} = 32.2, SD_{age} = 9.6) remained for the AV task, and 53 synaesthetes (41 female, 7 male, 5 undeclared, M_{age} = 29.8, SD_{age} = 9.0) and 53 controls (43 female, 10 male, M_{age} = 31.2, SD_{age} = 9.5) remained for the VV task.

3.1. Behavioural results

The matching procedure eliminated the difference in 75% detection likelihood threshold, as desired (AV: $t(102) = -0.122$, $p = 0.903$; VV: $t(103.98) = 0.144$, $p = 0.886$; see Supplementary Figure 5).

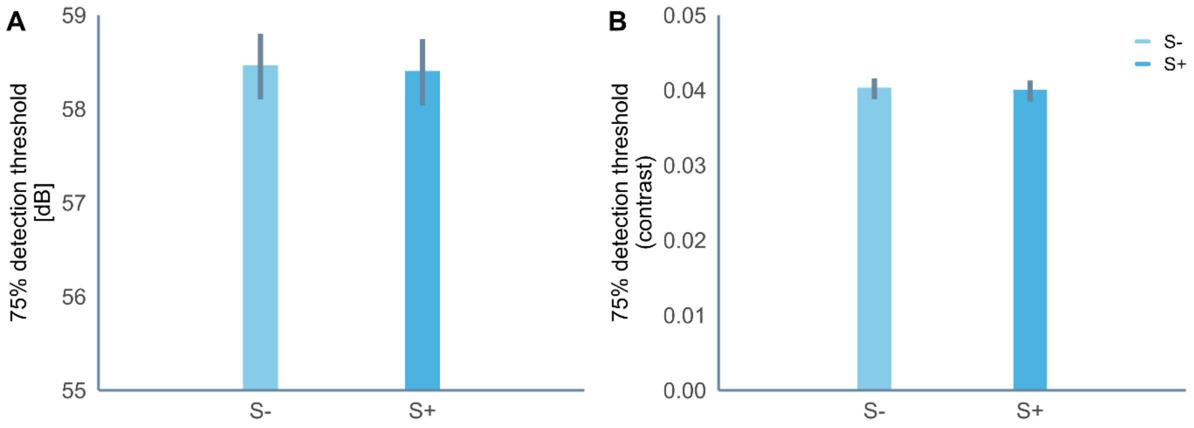

Supplementary Figure 5: 75%-likelihood detection thresholds for synaesthetes and controls matched on initial detection thresholds in **A** the auditory and **B** the visual modality. Error bars depict the standard error of the mean.

In the main task, the main effect of synaesthesia remained significant (AV: $F(1, 102) = 7.779$, $p = 0.006$; VV: $F(1, 104) = 21.003$, $p < 0.001$), and the interaction between synaesthesia and condition was significant in the VV task ($F(2.14, 222.47) = 4.430$, $p = 0.011$). The crucial comparison of reported detections in the no-target condition (i.e., the number of 'conditioned hallucinations') was not significant in either task (AV: $t(89.699) = 1.360$, $p = 0.177$; VV: $t(103.77) = -0.097$, $p = 0.923$; see Supplementary Figure 6). Average d' was significantly improved in synaesthetes in the VV task ($t(103.75) = -3.525$, df = 103.75, $p < 0.001$), but not the AV task ($t(100.05) = -1.35$, $p = 0.1801$).

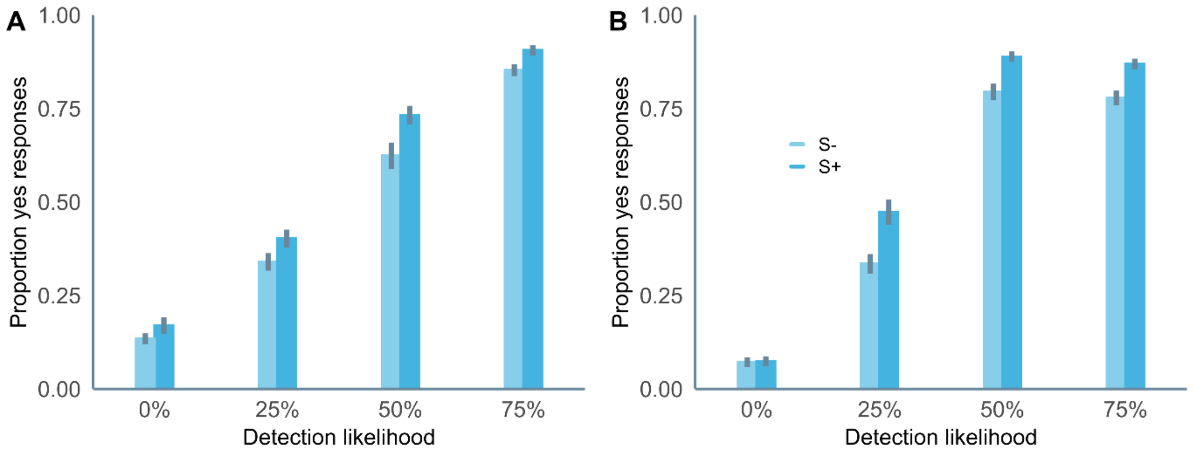

Supplementary Figure 6: Proportion of yes responses for synaesthetes and controls matched on initial detection thresholds in **A** the AV task and **B** the VV task. Error bars depict the standard error of the mean.

3.2. HGF results

The pattern of results pertaining to the belief trajectories also remained unchanged after matching, whereby only the stronger beliefs in the association of the cue and the target were significantly higher and more fixed in the AV task (X_1: $F(1, 102) = 2.232$, $p = 0.138$; X_2: $F(1,102) = 4.073$, $p = 0.046$; group × block interaction: $F(3.48, 354.61) = 3.539$, $p = 0.011$; X_3 $F(1, 102) = 0.044$, $p = 0.835$), while the full pattern of stronger beliefs in the target presence and in the association of the cue and the target remained significant in the VV task (X_1: $F(1, 104) = 14.519$, $p < 0.001$; X_2 $F(1, 104) = 13.763$, $p < 0.001$; X_3: $F(1, 104) = 0.541$ $p = 0.464$; see Supplementary Figure 7).

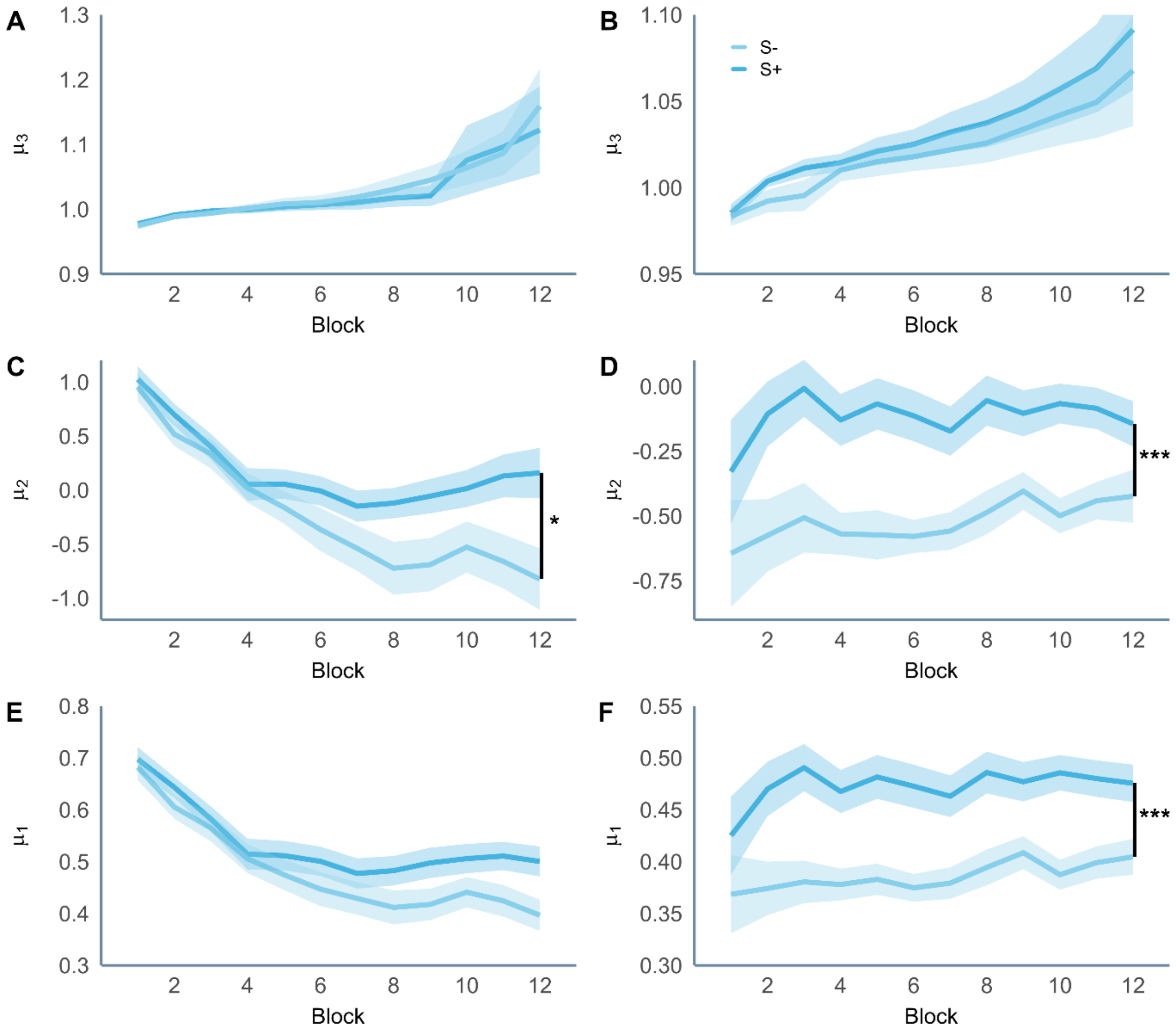

Supplementary Figure 7: Estimated belief trajectories for synaesthetes and controls per task, matched on initial detection thresholds : X_1 represents the belief that the conditioned stimulus was present or not on a given trial (AV task in **E**, VV task in **F**), X_2 represents the belief that the cue and the conditioned stimulus are associated (AV task in **C**, VV task in **D**), and X_3 represents the belief in the volatility of the association (AV task in **A**, VV task in **B**), where $\mu_{1\text{-}3}$ is the current belief or posterior at the corresponding level $X_{1\text{-}3}$. Shaded regions depict the standard error of the mean, asterisks refer to the main effect of synaesthesia. $p < 0.05$, ** $p < 0.01$, *** $p < 0.001$

Synaesthetes did not weight their priors differently relative to the sensory evidence, as quantified by parameter ν (AV: $t(100.71) = 0.653$, $p = 0.516$; VV: $t(99.618) = 0.583$, $p = 0.562$), nor did they differ in the stochasticity of their responses, indexed by parameter β^{-1} (AV: $t(100.87) = -0.213$, $p = 0.832$; VV: $t(98.112) = 1.448$, $p = 0.151$). They had somewhat decreased values for parameter ω_2, which captures attention to new information, in the VV task ($t(100.24) = 2.042$, $p = 0.044$) but not the AV task ($t(101.81) = 0.202$, $p = 0.840$; see Supplementary Figure 8).

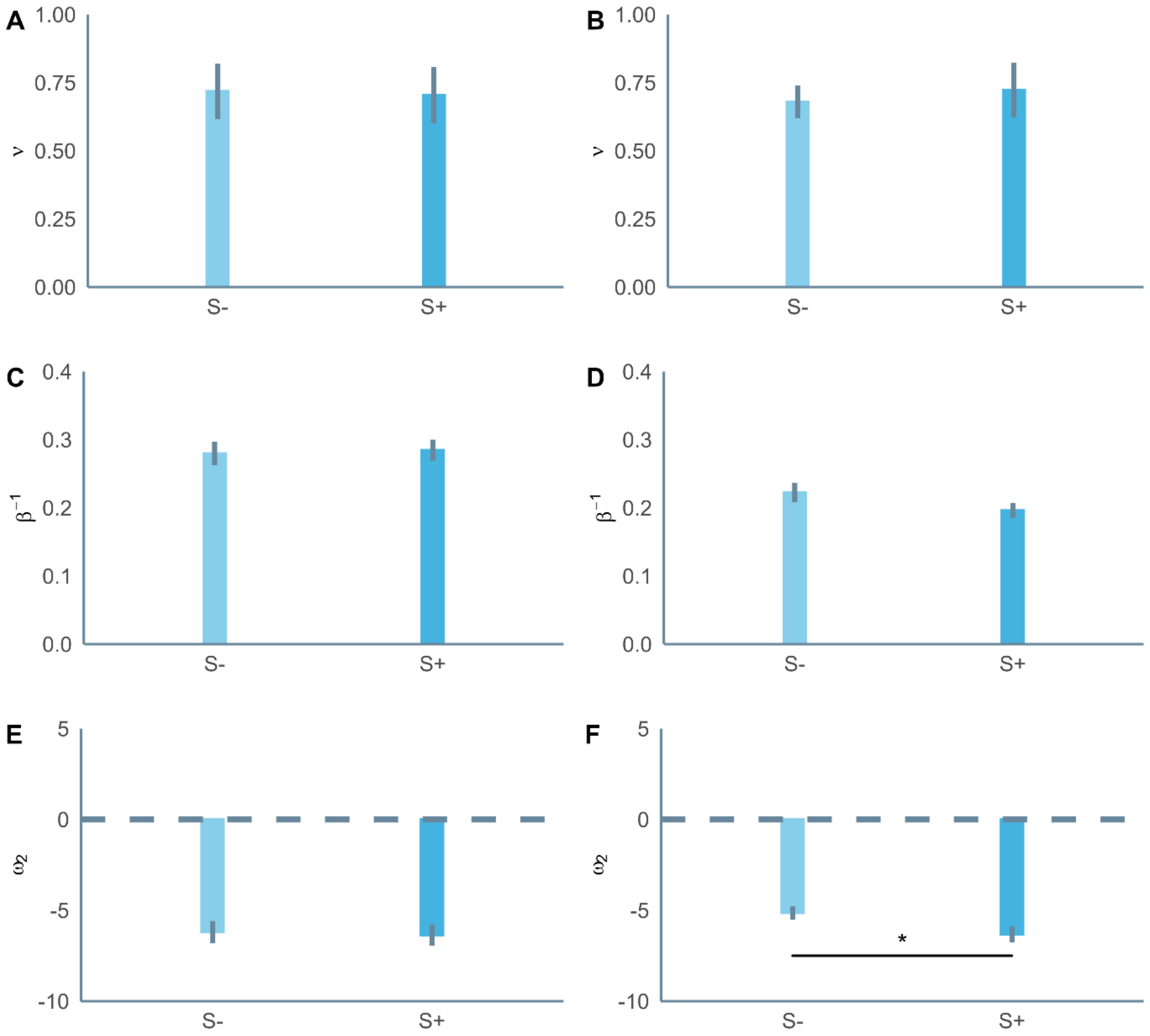

Supplementary Figure 8: Estimated parameters ν (indexing the relative weight of the prior compared to the sensory evidence; AV task in **A**, VV task in **B**), β^{-1} (indexing stochasticity of response; AV task in **C**, VV task in **D**), and ω_2 (indexing the estimate of baseline environmental volatility; AV task in **E**, VV task in **F**) for synaesthetes and controls matched on initial detection thresholds. Error bars depict the standard error of the mean. p <0.05, ** p <0.01, *** p <0.001

4. Signal detection analysis of performance

Performance in this task can be expressed using signal detection measures. Here, the signal detection measures d' and criterion are estimated per block using the R package psycho, and subsequently averaged across blocks.

Synaesthetes' d' was significantly higher than controls' in the VV task ($t(148.36) = 2.572$, $p = 0.011$), but not the AV task ($t(145.67) = 0.440$, $p = 0.661$; see Supplementary Figure 9A and B). While the criterion tended to be lower in synaesthetes compared to controls, the differences were not significant

in either the AV task (t(125.76) = -0.532, p = 0.596) or the VV task (t(142.22) = -1.787, p = 0.076; see Supplementary Figure 9C and D).

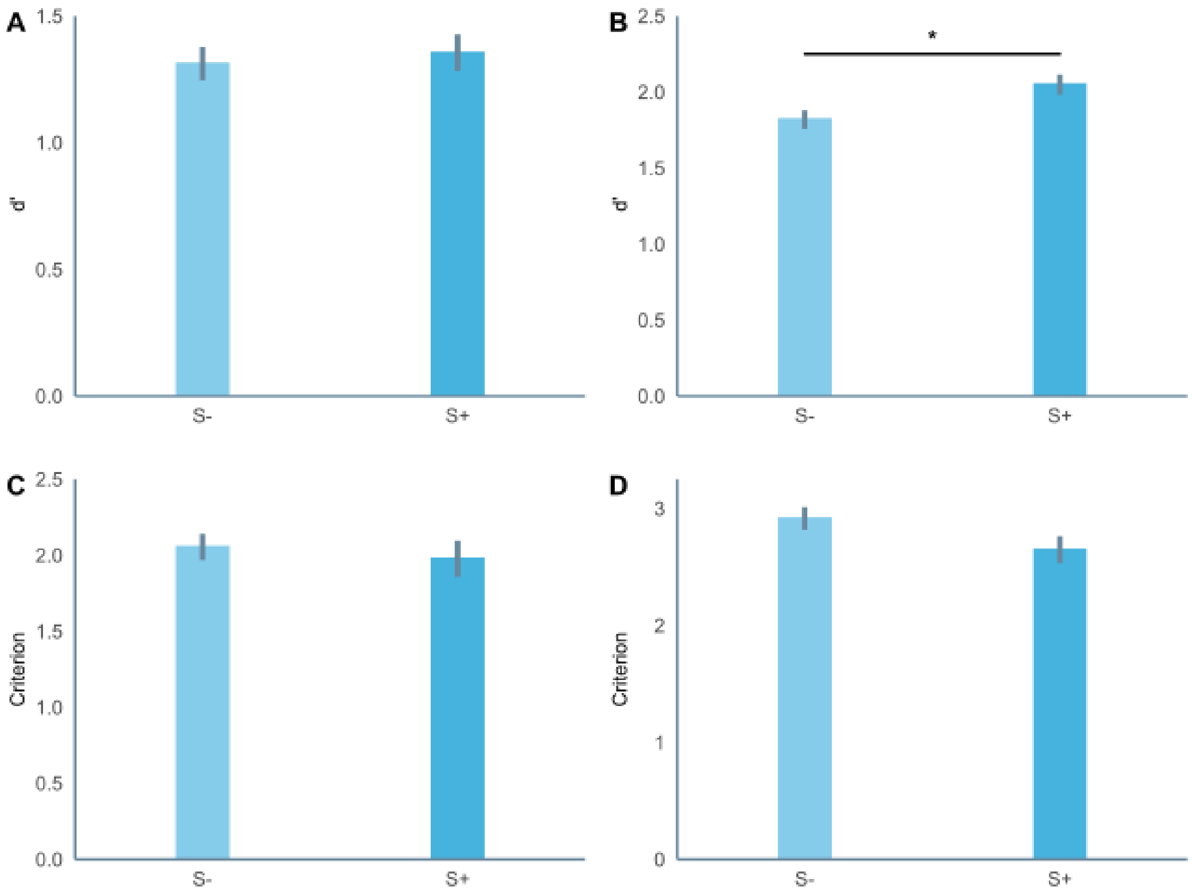

Supplementary Figure 9: Signal detection measures d' averaged across blocks (AV task in **A**, VV task in **B**), and criterion averaged across blocks (AV task in **C**, VV task in **D**) for synaesthetes and controls. Error bars depict the standard error of the mean. p <0.05, ** p <0.01, *** p <0.001

5. Performance over time

The evolution of the detection responses throughout the experiment are shown in Supplementary Figure 10. In a three-way ANOVA, there was a main effect of synaesthesia in both tasks (AV: F(1,156) = 7.050, p = 0.009; VV: F(1, 153) = 13.311 p < 0.001), and an interaction between synaesthesia and block in the AV task (F(6.16, 960.54) = 2.510, p = 0.019). For the evolution of d' over time, the two-way ANOVA showed a significant main effect of synaesthesia in the VV (F(1,153) = 6.623, p = 0.011), but not the AV task (F(1,156) = 0.189, p = 0.664).

The fact that synaesthetes have an overall advantage in the VV task is noteworthy given that our thresholding procedure should have made the task equally difficult for everyone. Supplementary Figure 10 suggests that the expected threshold level of performance was broadly maintained in block

1 but had sustained improvement from block 2 onwards (e.g., such that the 50% threshold generates ~70-80% correct performance). For the AV task, the perceptual advantage was either apparent on the initial thresholds (but not the task) or was present in the task (when thresholds are matched) but not both. Either way, synaesthetes had some perceptual advantage over controls.

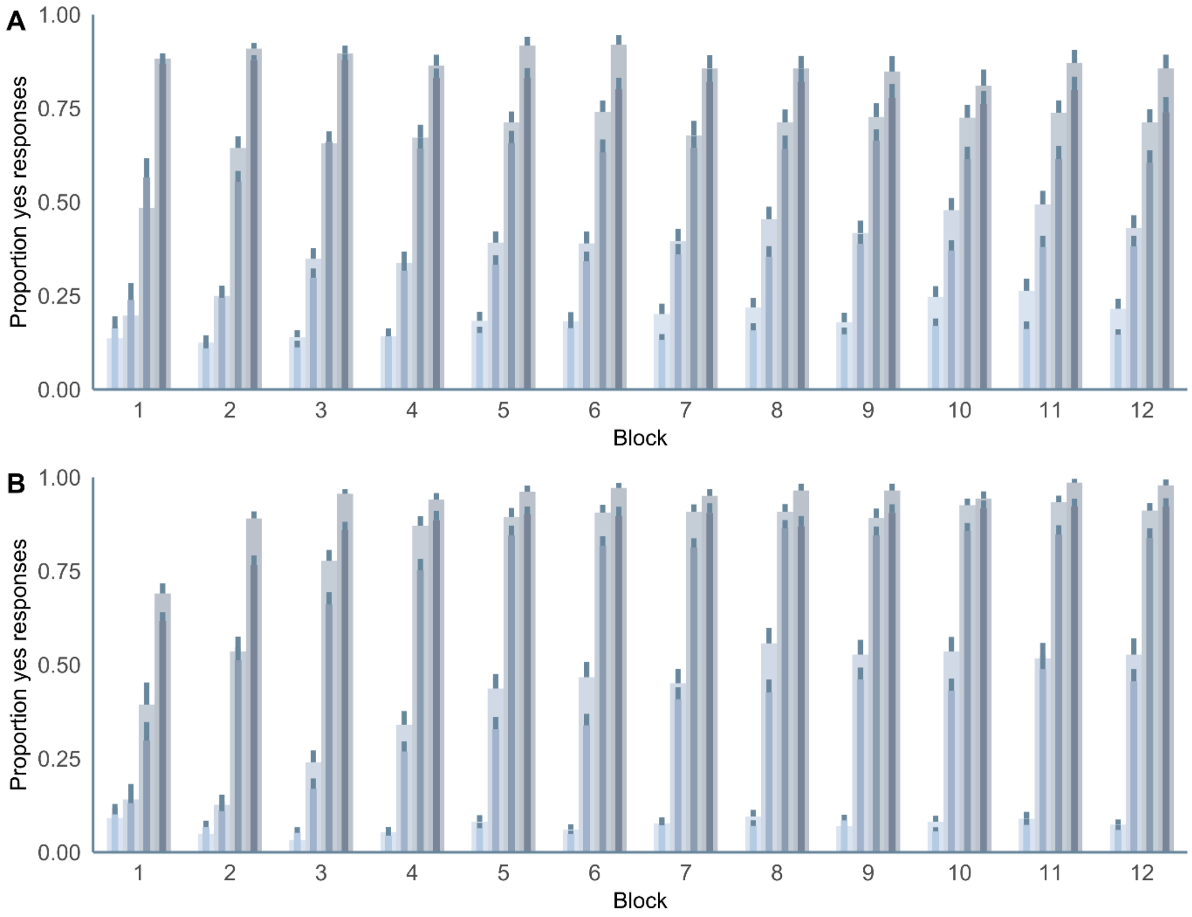

Supplementary Figure 10: Evolution of the detection reports for synaesthetes and controls per condition and block in **A** the AV task and **B** the VV task. Synaesthetes are depicted in wider and higher-transparency bars. Error bars depict the standard error of the mean.

6. Model comparison

The original model employed in the discovery experiment was fit using participants' expected detection rates as the stimulus intensity inputs, whereas subsequent studies have reported improved results when the fit was based instead on the empirical grand mean detection rates across participants[1]. Model fit quality for both versions of the HGF was assessed by means of model inversion (10000 simulations) and a Bayesian model comparison using the spm_BMS function (equivalent to a random effects analysis[6]). Based on the model inversion results, there was no significant difference in the AV task (t(312.15) = -0.663, p = 0.508), and a significant improvement for the empirical value model in the VV task (t(307.68) = 3.090, p = 0.002). The Bayesian model comparison favoured the empirical

value model in both tasks (see Supplementary Figure 11).

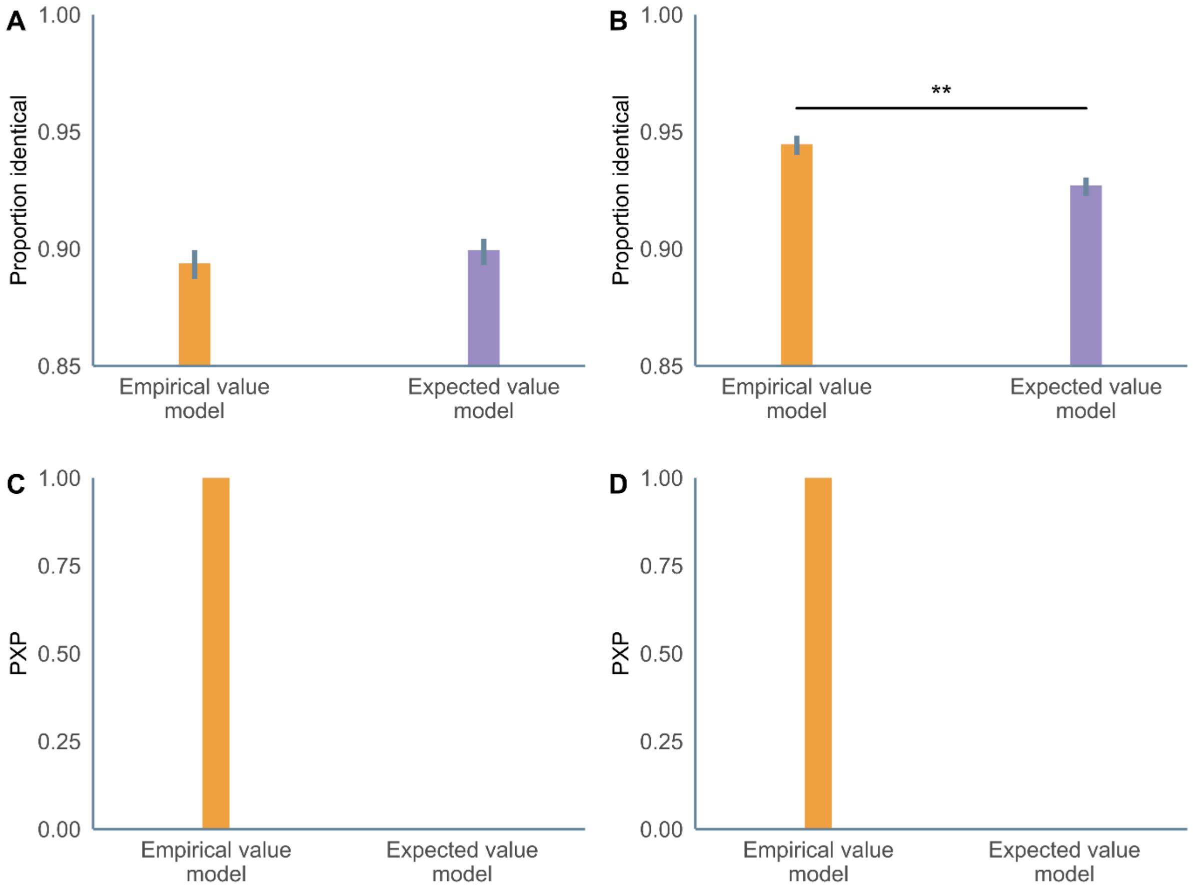

Supplementary Figure 11: Model comparison for two HGF model versions pooling all data: the original model employed in the discovery experiment fit using participants' target detection rates as the stimulus intensity inputs (expected value model) and a second HGF model fit using instead the empirical grand mean detection rates across participants (empirical value model). Fit quality was quantified using the number of identical responses after model inversion in **A** the AV task and **B** the VV task and using Bayesian model selection in **C** the AV task and **D** the VV task. Error bars depict the standard error of the mean.

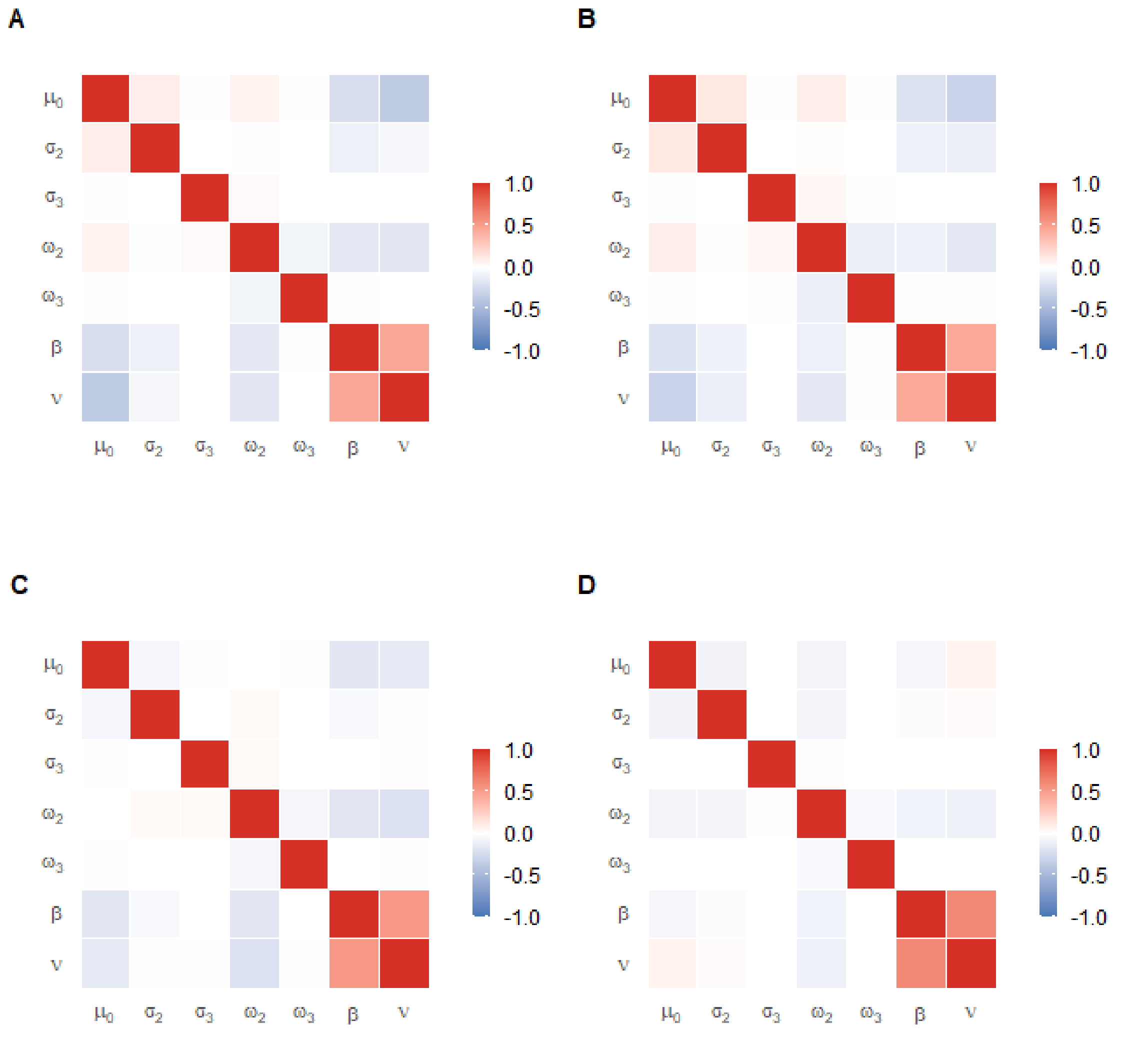

Supplementary Figure 12: Parameter identifiability for two HGF model versions pooling all data: the original model employed in the discovery experiment fit using participants' target detection rates as the stimulus intensity inputs (expected value model) and a second HGF model fit using instead the empirical grand mean detection rates across participants (empirical value model). Correlation matrices for the estimated parameters for the expected value model in **A** the AV and **B** the VV task, and for the empirical value model in **C** the AV task and **D** the VV task.

Irrespective of the fitting procedure, the main findings were robust to this decision (see Supplementary Figure 13). The pattern found for the belief trajectories was consistent overall with those estimated using the alternative fitting procedure, whereby in the AV task only the interaction effects at X_1 and X_2 were significant (X_1: $F(1, 156) = 2.879$, $p = 0.092$; $F(5.08, 792.41) = 2.633$, $p = 0.022$; X_2: $F(1, 156) = 3.722$, $p = 0.056$; group × block interaction: $F(5.08, 792.41) = 3.082$, $p = 0.015$; X_3: $F(1, 156) = 0.089$, $p = 0.766$) while in the VV task the main effects at X_1 and X_2 were significant (X_1: $F(1, 153) = 15.592$, $p < 0.001$; X_2: $F(1, 153) = 14.133$, $p < 0.001$; $F(1, 153) = 0.207$, $p = 0.649$).

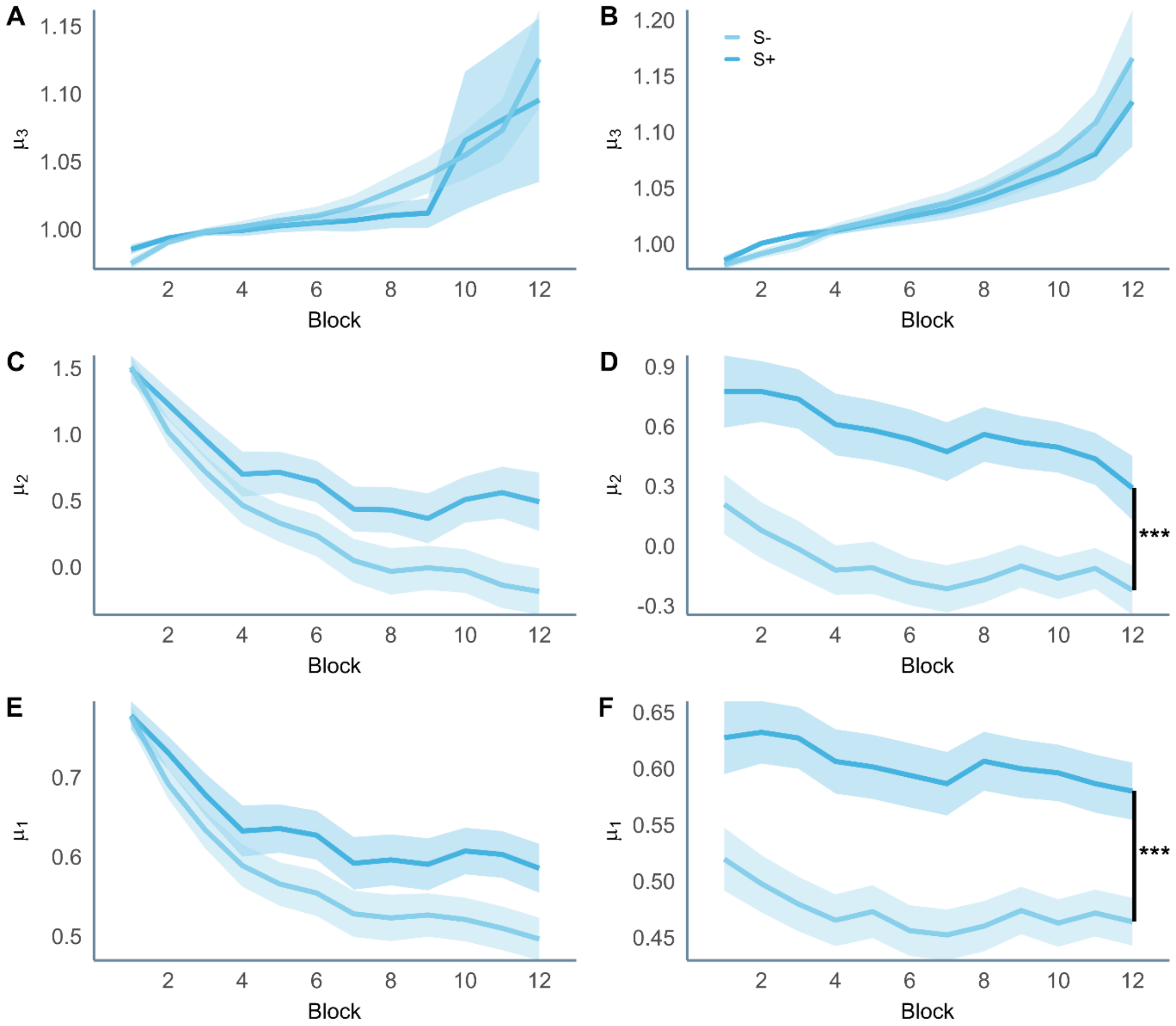

Supplementary Figure 13: Estimated belief trajectories for synaesthetes and controls per task using the expected value model : X_1 represents the belief that the conditioned stimulus was present or not on a given trial (AV task in **E**, VV task in **F**), X_2 represents the belief that the cue and the conditioned stimulus are associated (AV task in **C**, VV task in **D**), and X_3 represents the belief in the volatility of the association (AV task in **A**, VV task in **B**) , where $\mu_{1\text{-}3}$ is the current belief or posterior at the corresponding level $X_{1\text{-}3}$. Shaded regions depict the standard error of the mean, asterisks refer to the main effect of synaesthesia. p <0.05, ** p <0.01, *** p <0.001

Consistent with the main results using the alternative fitting procedure, there were no significant differences in parameters ν, β^{-1} and ω_2 in the AV task (ν: t(135.2) = 0.680, p = 0.498; β^{-1}: t(131.55) = -0.191, p = 0.849; ω_2: t(144.5) = -1.444, p = 0.151). However, the differences observed in the β^{-1} and ω_2 parameters in the VV task were significant using the expected value model (ν: t(146.43) = 1.863, p = 0.064; β^{-1}: t(142.4) = -3.266, p = 0.001; ω_2: t(140.79) = -3.110, p = 0.002; see Supplementary Figure 14).

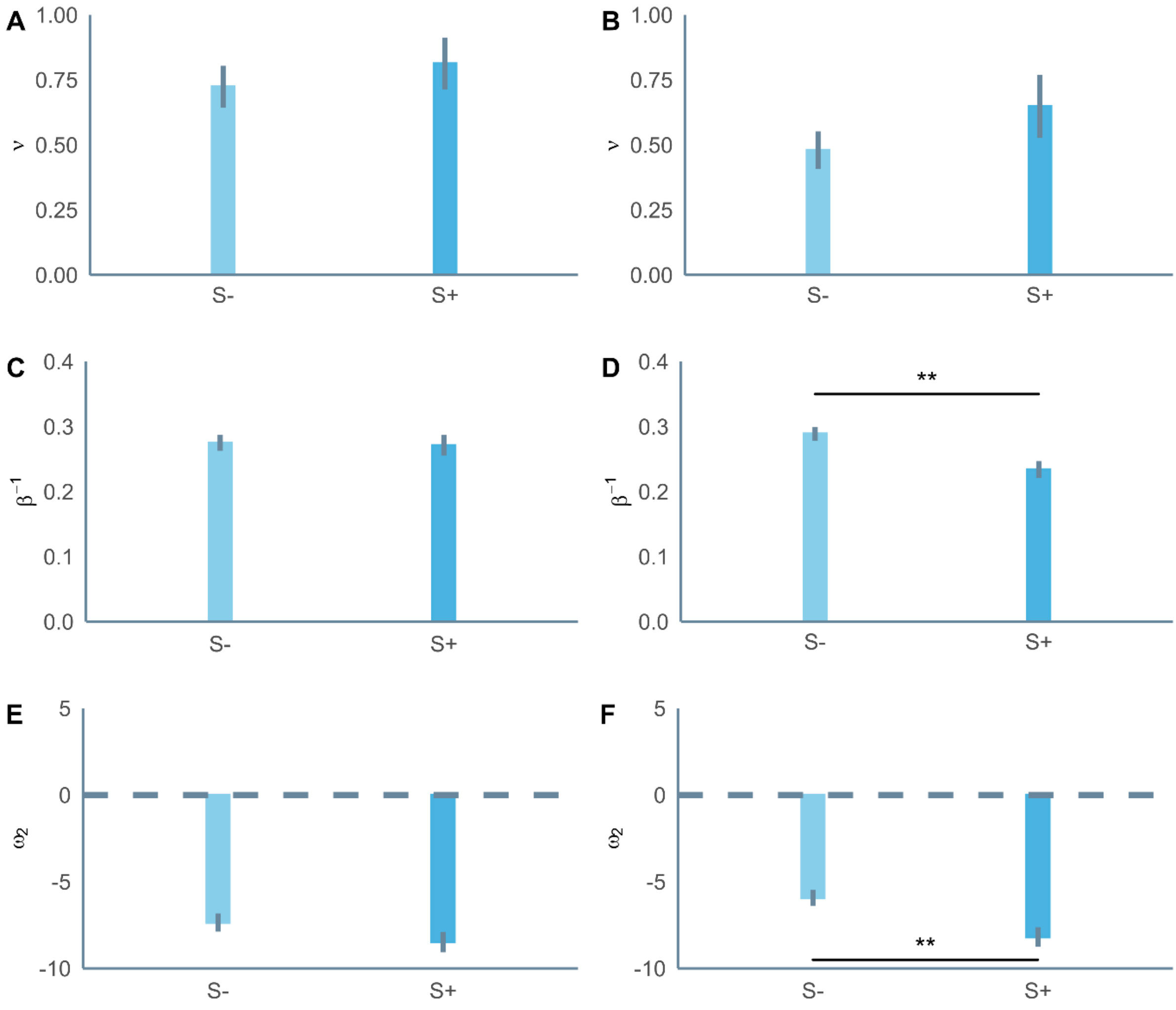

Supplementary Figure 14: Estimated parameters ν (indexing the relative weight of the prior compared to the sensory evidence; AV task in **A**, VV task in **B**), β^{-1} (indexing stochasticity of response; AV task in **C**, VV task in **D**), and ω_2 (indexing the estimate of baseline environmental volatility; AV task in **E**, VV task in **F**) for synaesthetes and controls using the expected value model. Error bars depict the standard error of the mean. p <0.05, ** p <0.01, *** p <0.001

7. Individual differences within synaesthesia

We explored individual differences within the synaesthesia group (see Supplementary Table 1) by evaluating the effect of synaesthesia intensity, quantified as 1) the number of types of synaesthesia and 2) the degree of externalisation of the synaesthetic experience as per the Coloured Letters and Numbers (CLaN) localisation subscale. In this context, it should be noted that both proxies of synaesthesia intensity were correlated within the sample who completed the AV task ($r = 0.248$, $p = 0.001$), though not significantly within the sample who completed the VV task ($r = 0.124$, $p = 0.106$).

Supplementary Table 1: Synaesthete sample characterization - prevalence of each type of synaesthesia

	**AV (n = 66)**	**VV (n = 70)**
**Language - colour**	64	67
**Language - taste**	8	12
**Language - touch**	8	10
**Visualised sensations**	47	56
**Sequence-space synaesthesia**	42	45
**Ordinal linguistic personification**	34	38
**Smell/taste experiences**	5	8
**Tickertape**	26	30
**Mirror- touch**	17	22
**Hearing motion**	23	31
**Auditory - visual**	32	41
**Mean number of synaesthesia types**	4 (SD = 2)	5 (SD = 2)
**CLaN localization score**	18.4 (SD = 5.9)	18.8 (SD = 5.8)

Neither of these metrics were significantly correlated with the key measures of interest in either task (rate of conditioned hallucinations, and parameters ν, β^{-1} and ω_2, see Supplementary Figures 16 and 18) or with the detection thresholds in either modality (number of types: auditory: $\rho_s = -0.184$, $p = 0.139$; visual: $\rho_s = -0.034$, $p = 0.777$; CLaN: auditory: $\rho_s = -0.084$, $p = 0.536$; visual: $\rho_s = 0.071$, $p = 0.600$). The number of types of synaesthesia did not modulate the belief trajectories in the AV task (X_1: $F(8,57) = 0.566$, $p = 0.801$; X_2: $F(8, 57) = 0.640$, $p = 0.741$; X_3: $F(8, 57) = 0.698$, $p = 0.691$), while in the VV task the volatility estimate and its learning rate were lower in synaesthetes with more types (X_1: $F(9, 60) = 0.463$, $p = 0.894$, X_2: $F(9, 60) = 0.479$, $p = 0.883$, X_3: $F(9, 60) = 2.348$, $p = 0.024$; group × block interaction: $F(9.19, 61.26) = 4.264$, $p < 0.001$; see Supplementary Figure 15). Synaesthetes with higher CLaN localization scores updated their beliefs in the target presence and its association with the cue more slowly, and concurrently had higher volatility estimates which increased more readily in the AV task (X_1: $F(20,36) = 0.900$, $p = 0.589$, group × block interaction: $F(98.36, 177.05) = 1.421$, $p = 0.022$; X_2: $F(20,36) = 1.209$, $p = 0.302$, group × block interaction: $F(92.01, 165.63) = 2.840 < 0.001$; X_3: $F(20, 36) = 8.362$, $p < 0.001$; group × block interaction: $F(22.52, 40.54) = 7.165$, $p < 0.001$). In the VV task they likewise had higher volatility estimates which increase more readily, yet showed no significant differences in any of the lower-level belief trajectories (X_1: $F(22, 34) = 0.556$, $p = 0.925$; X_2: $F(22, 34) = 0.547$, $p = 0.930$, X_3: $F(22, 34) = 10.955$, $p < 0.001$; group × block interaction: $F(23.18, 35.82) = 8.468$, $p < 0.001$; see Supplementary Figure 17).

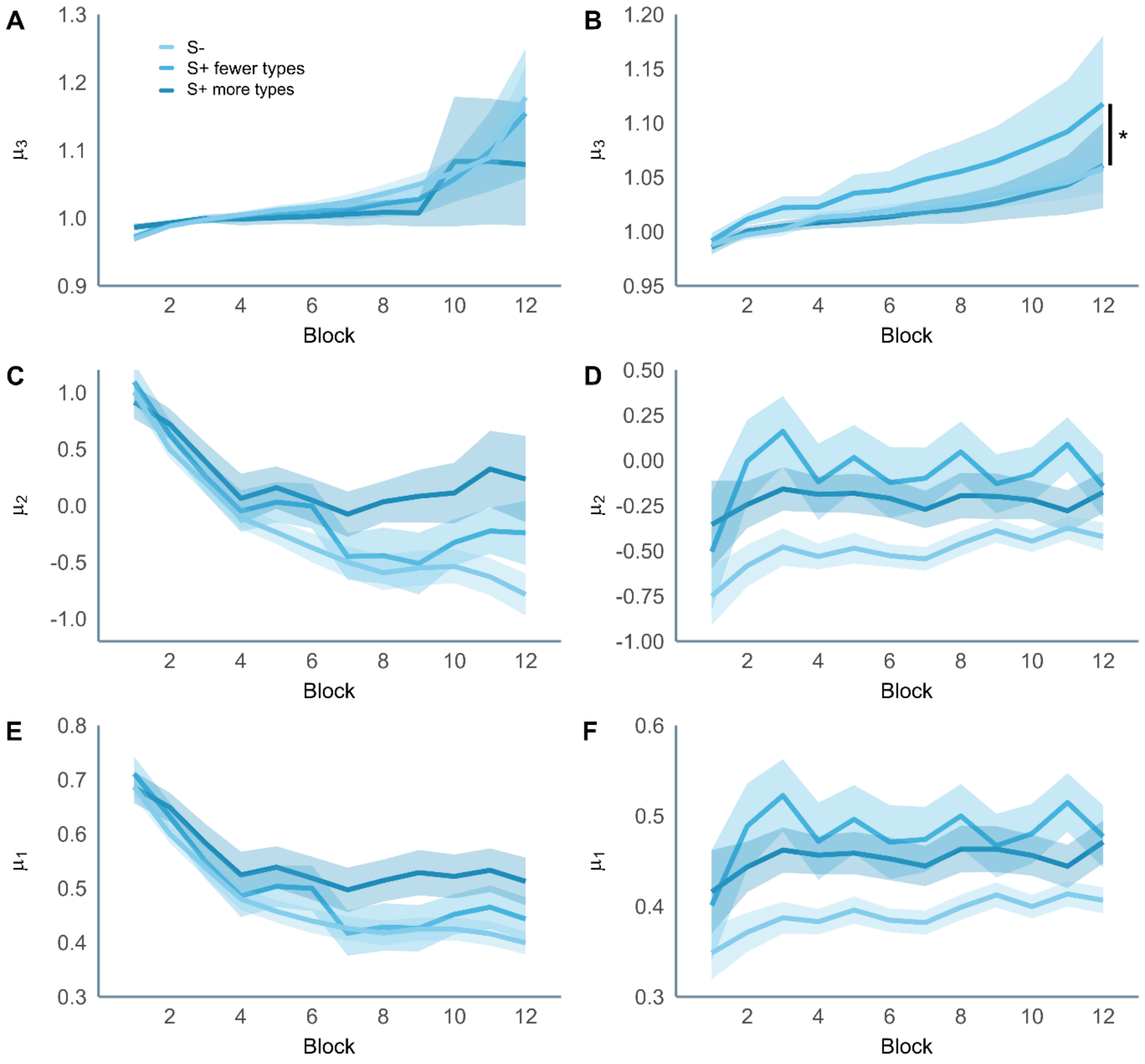

Supplementary Figure 15: Estimated belief trajectories per task for synaesthetes with a high and low number of synaesthesia types as per a median split (more and fewer than 4 types respectively – n_{high} = 26, n_{low} = 28 in the AV task, and n_{high} = 28, n_{low} = 27 in the VV task) and controls: X_1 represents the belief that the conditioned stimulus was present or not on a given trial (AV task in **E**, VV task in **F**), X_2 represents the belief that the cue and the conditioned stimulus are associated (AV task in **C**, VV task in **D**), and X_3 represents the belief in the volatility of the association (AV task in **A**, VV task in **B**) , where $\mu_{1\text{-}3}$ is the current belief or posterior at the corresponding level $X_{1\text{-}3}$. Shaded regions depict the standard error of the mean, asterisks refer to the main effect of synaesthesia.

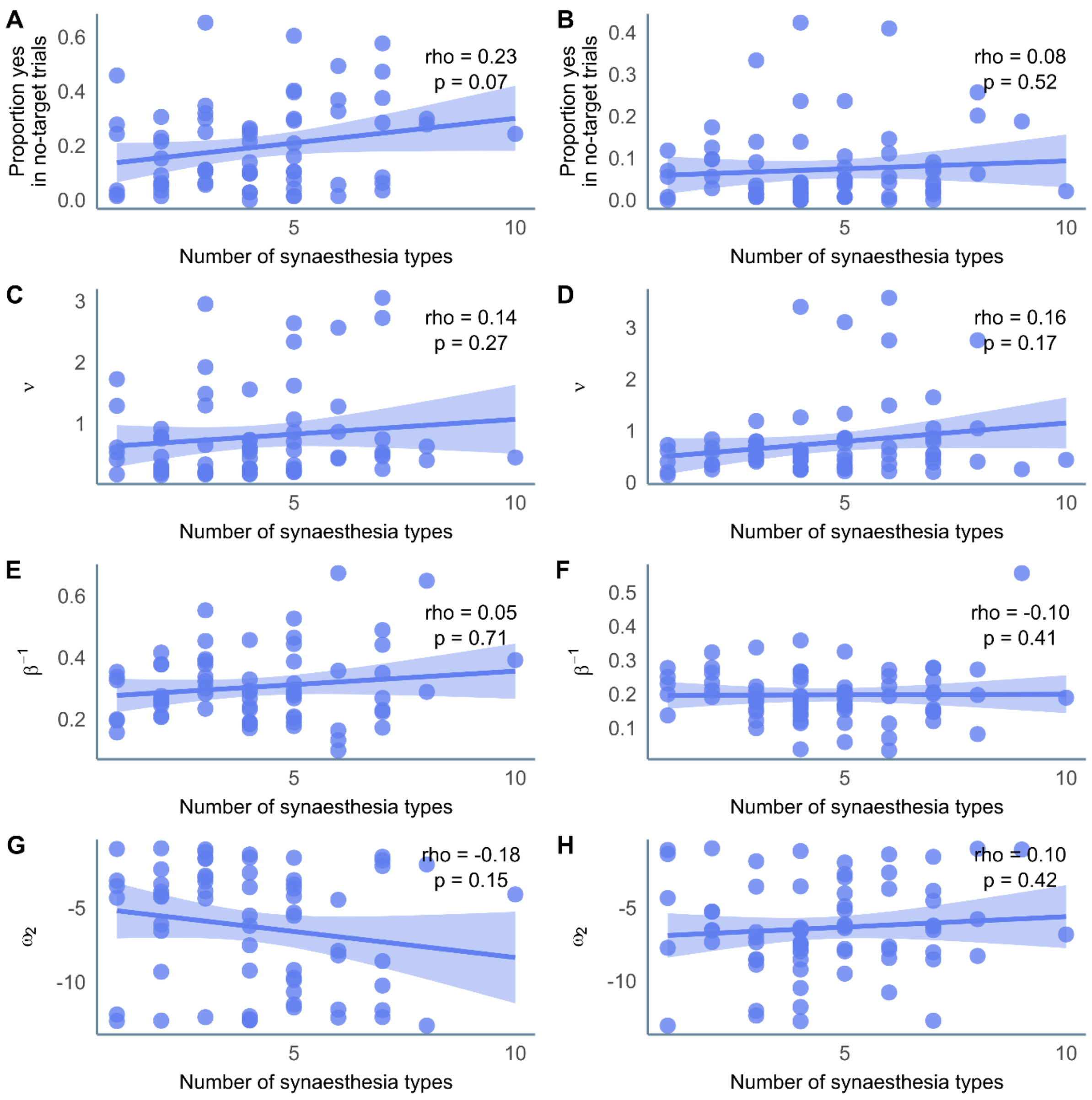

Supplementary Figure 16: Correlation between the number of synaesthesia types and the key measures of interest: the rate of conditioned hallucinations (AV task in **A**, VV task in **B**), estimated parameter ν (indexing the relative weight of the prior compared to the sensory evidence; AV task in **C**, VV task in **D**), β^{-1} (indexing stochasticity of response; AV task in **E**, VV task in **F**), and ω_2 (indexing the estimate of baseline environmental volatility; AV task in **G**, VV task in **H**).

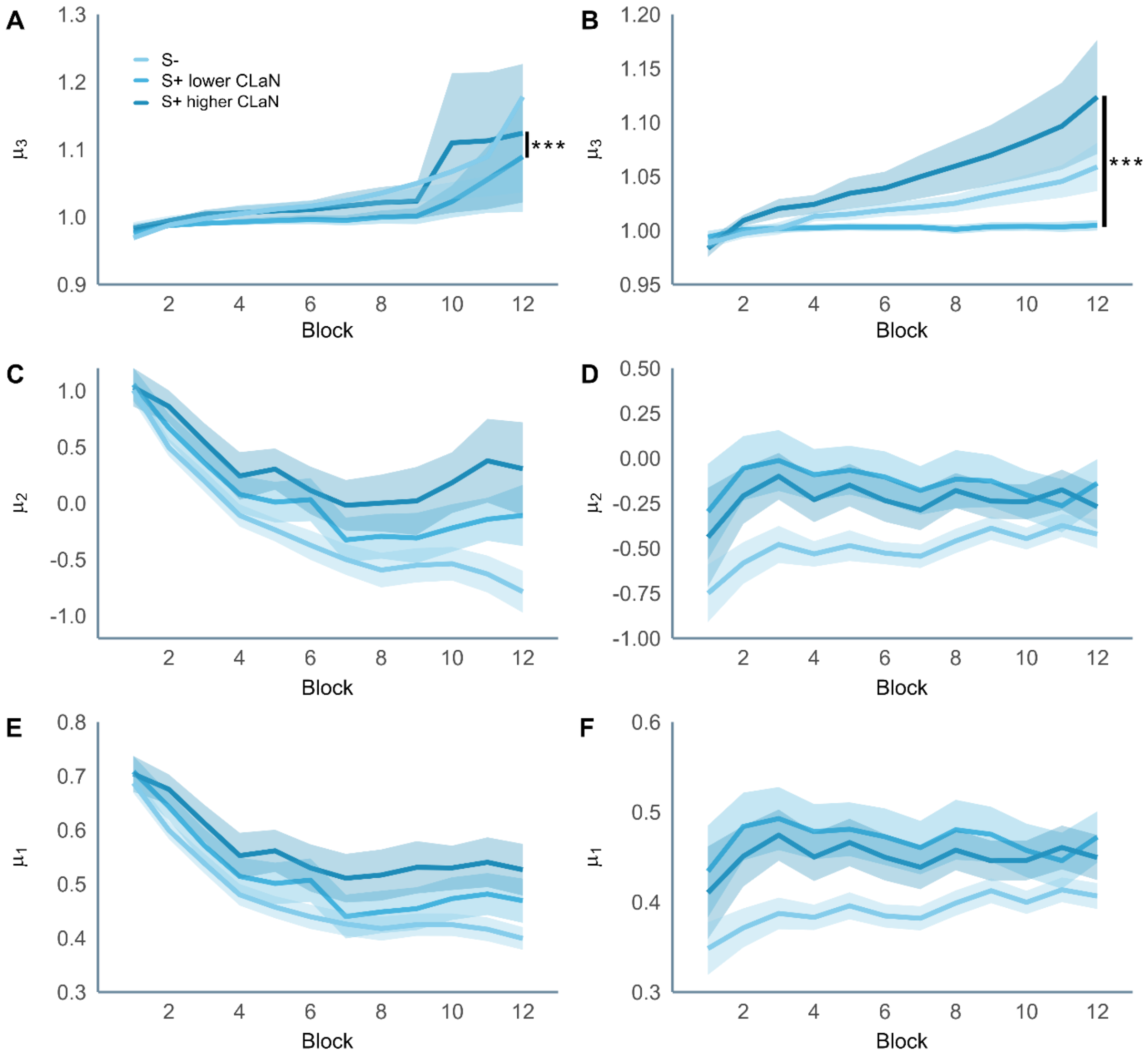

Supplementary Figure 17: Estimated belief trajectories per task for synaesthetes with a high and low CLaN localisation subscale score as per a median split (a score above and below 19 respectively – n_{high} = 26, n_{low} = 31 in the AV task, and n_{high} = 31, n_{low} = 34 in the VV task) and controls: X_1 represents the belief that the conditioned stimulus was present or not on a given trial (AV task in **E**, VV task in **F**), X_2 represents the belief that the cue and the conditioned stimulus are associated (AV task in **C**, VV task in **D**), and X_3 represents the belief in the volatility of the association (AV task in **A**, VV task in **B**) , where $\mu_{1\text{-}3}$ is the current belief or posterior at the corresponding level $X_{1\text{-}3}$. Shaded regions depict the standard error of the mean, asterisks refer to the main effect of synaesthesia.

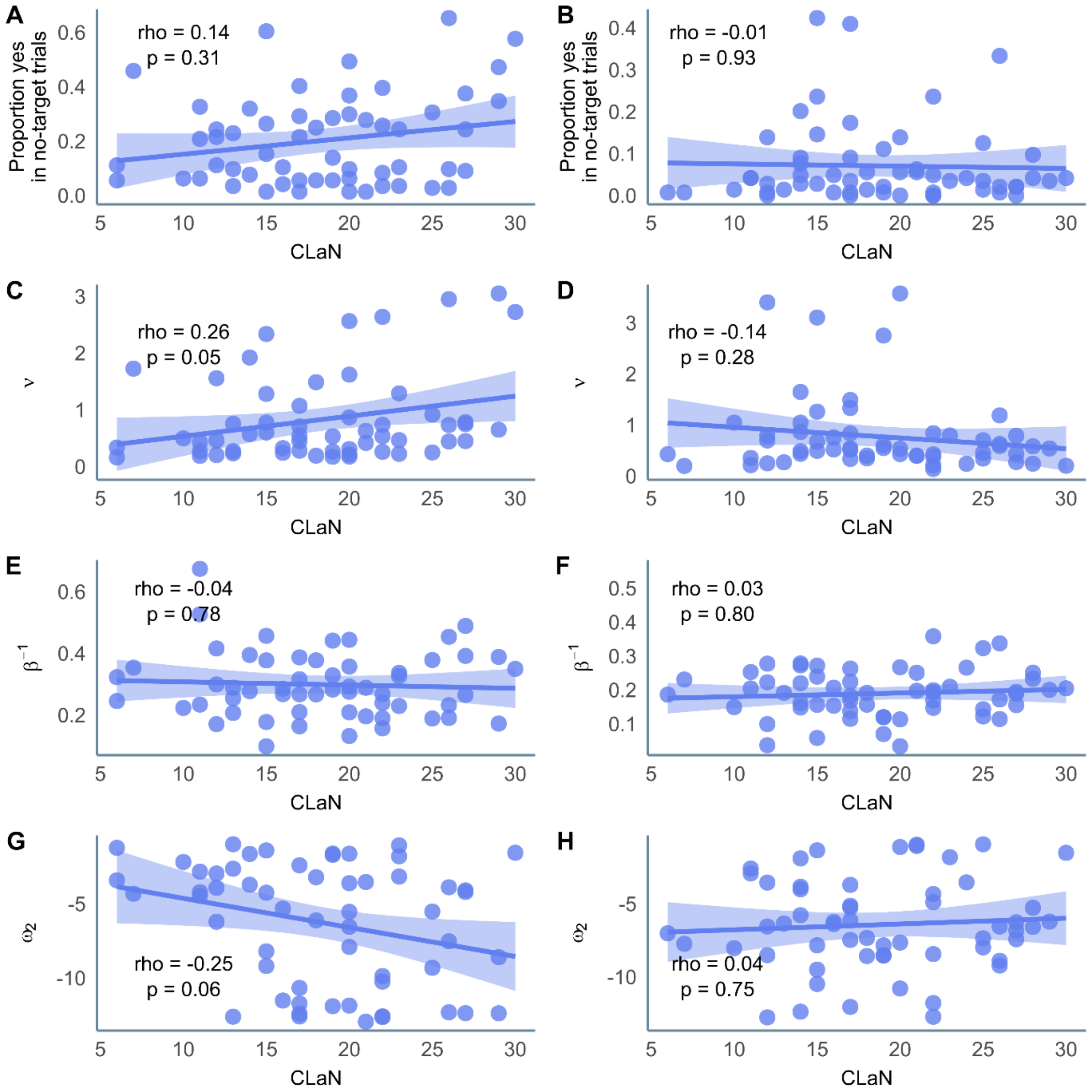

Supplementary Figure 18: Correlation between the localisation scale of the CLaN questionnaire and the key measures of interest: the rate of conditioned hallucinations (AV task in **A**, VV task in **B**), estimated parameter ν (indexing the relative weight of the prior compared to the sensory evidence; AV task in **C**, VV task in **D**), β^{-1} (indexing stochasticity of response; AV task in **E**, VV task in **F**), and ω_2 (indexing the estimate of baseline environmental volatility; AV task in **G**, VV task in **H**).

We additionally explored the effect of the sensory modalities involved in the synaesthesia type. As the especially designed VV task aimed to target grapheme-colour synaesthesia, we selected participants with types of synaesthesia involving audition: hearing motion synaesthesia (HMS) and auditory to visual synaesthesia (AVS). Synaesthetes with HMS had increased rates of conditioned hallucinations in both the AV ($t(36.473) = -2.187$, $p=0.035$) and the VV task ($t(45.578) = 2.115$, $p = 0.040$) relative to synaesthetes without HMS with no differences in ν, β^{-1}, or ω_2 in either task (AV: ν: $t(42.195) = 1.970$, $p = 0.055$; β^{-1}: $t(30.196) = 0.486$, $p = 0.630$; ω_2: $t(44.169) = -1.898$, $p = 0.064$; VV: ν: t(52.466)

= 1.228, p = 0.225; β^{-1} t(49.493) = 0.416, p = 0.679, ω_2: t(63.22) = 0.464, p = 0.644; see Supplementary Figure 20). This does not survive correction for multiple comparisons, although there is other suggestive yet not significant evidence that this group performs differently: they show stronger and more fixed beliefs in the presence of the tone and in the association of the checkerboard with the tone, with other synaesthetes being intermediate between this group and the controls in the AV task (X_1: F(1, 64) = 2.321, p = 0.133; X_2: F(1,64) = 2.686, p = 0.106; X_3 : F(1, 64) = 1.334, p = 0.565), though this is also the case to a lesser degree in the VV task (X_1: F(1, 68) = 1.157, p = 0.286, X_2: F(1, 68) = 0.808, p = 0.372, X_3: F(1, 68) = 0.435, p = 0.512; see Supplementary Figure 19).

The opposite profile of synaesthesia, where tones trigger visual experiences such as colour, was not linked to any significant differences compared to all other synaesthetes who lack this type. Synaesthetes with AVS did not show increased rates of conditioned hallucinations in either the AV (t(63.838) = -0.184, p = 0.854) or VV task (t(57.834) = 0.311, p = 0.757), and there were no differences in parameters in either task relative to all other synaesthetes (AV: ν: t(63.965) = -0.127, p = 0.899; β^{-1}: t(53.531) = 0.130, p = 0.897; ω_2: t(63.846) = -1.081, p = 0.284; VV: ν: t(53.749) = 0.826, p = 0.413; β^{-1}: t(67.041) = 0.786, p = 0.435; ω_2: t(58.203) = 1.370, p = 0.176; see Supplementary Figure 22). The belief trajectories were likewise very similar to those of all other synaesthetes in the AV task (X_1: F(1,64) = 0.242, p = 0.624; X_2: F(1, 64) = 0.431, p = 0.514, X_3: F(1, 64) = 0.662, p = 0.419), while, if anything, they appear to have weaker beliefs in the presence of the colour and in the association of the checkerboard and the colour relative to other synaesthetes in the VV task (X_1: F(1,68) = 0.898, p = 0.347; X_2: F(1, 68) = 1.308, p = 0.257, X_3: F(1, 68) = 0.532, p = 0.468; see Supplementary Figure 21). While this is perhaps unexpected, it should be noted that all but three synaesthetes have grapheme-colour synaesthesia, i.e., individuals with auditory-to-visual synaesthesia in addition to grapheme-colour synaesthesia have fewer visual-visual conditioned hallucinations relative to those who have primarily visual-visual experiences.

Detection thresholds were not significantly different in these specific types of synaesthesia relative to all other synaesthetes in either modality (HMS: AV: t(48.783) = -1.669, p = 0.101; VV: t(66.223) = -0.641, p = 0.524; AVS: AV: t(63.324) = 0.874, p = 0.385; VV: t(67.977) = 0.065, p = 0.948).

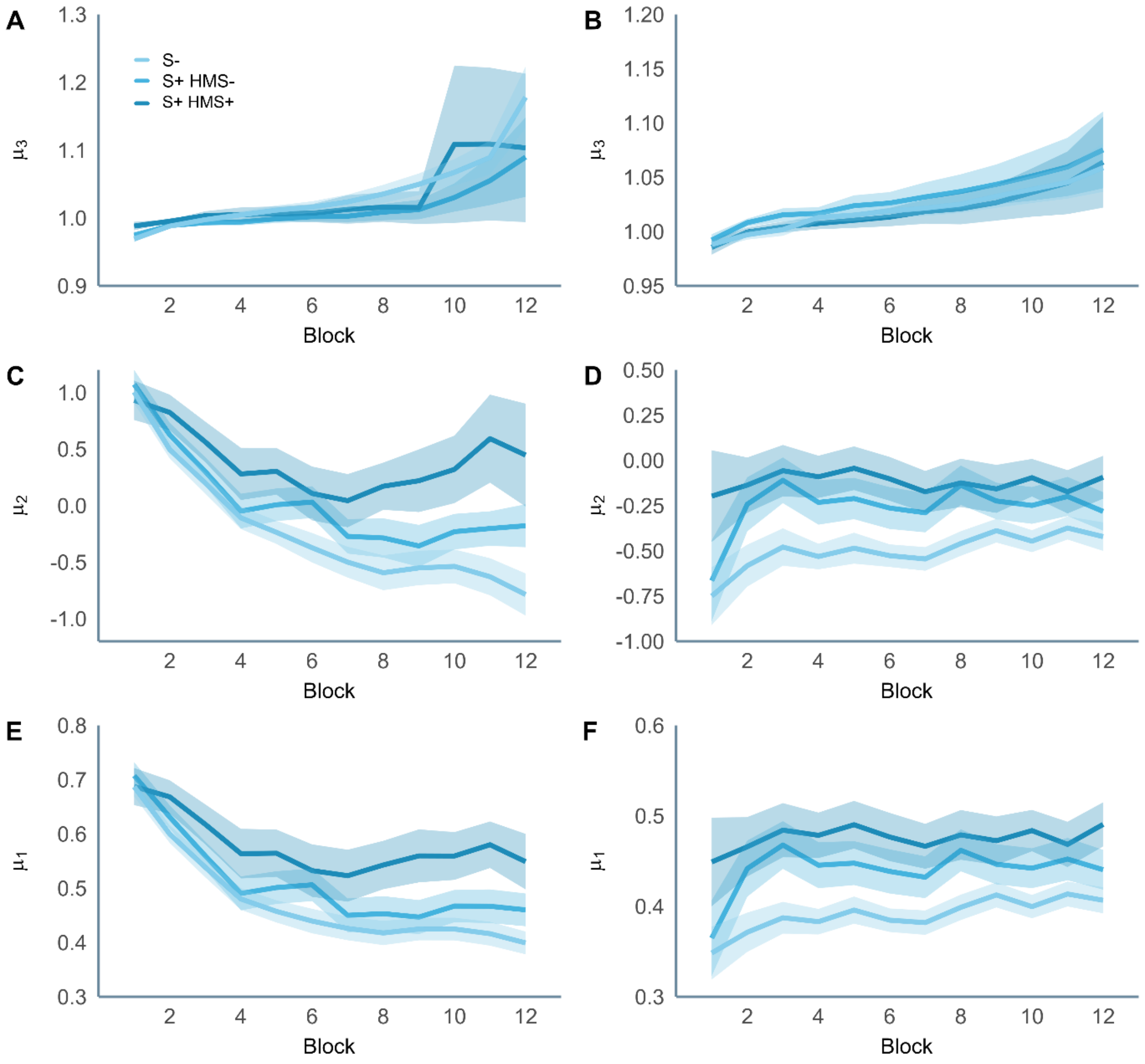

Supplementary Figure 19: Estimated belief trajectories per task for synaesthetes with hearing motion synaesthesia (n = 23 in the AV task and n = 31 in the VV task), all other synaesthetes, and controls: X_1 represents the belief that the conditioned stimulus was present or not on a given trial (AV task in **E**, VV task in **F**), X_2 represents the belief that the cue and the conditioned stimulus are associated (AV task in **C**, VV task in **D**), and X_3 represents the belief in the volatility of the association (AV task in **A**, VV task in **B**) , where $\mu_{1\text{-}3}$ is the current belief or posterior at the corresponding level $X_{1\text{-}3}$. Shaded regions depict the standard error of the mean.

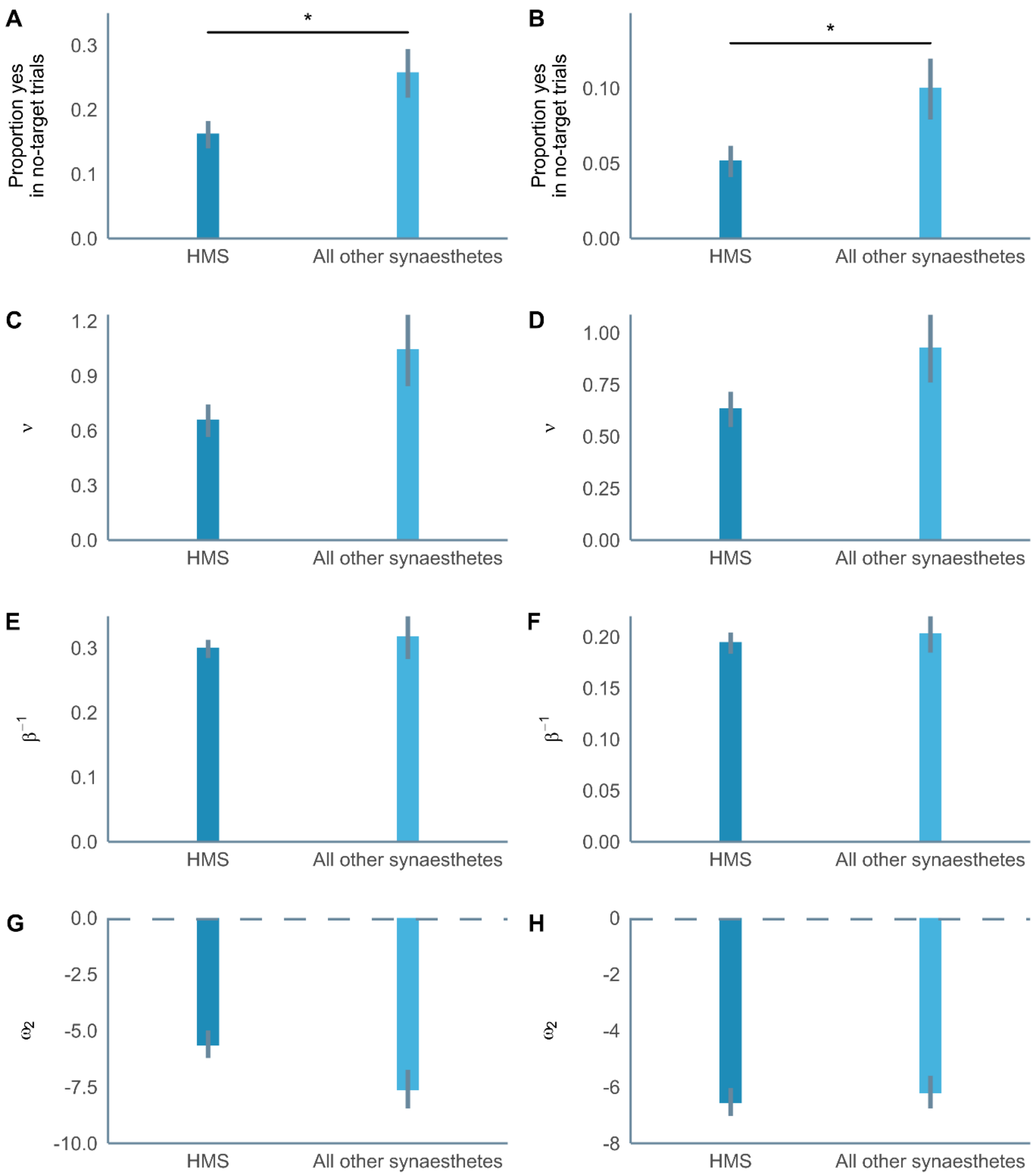

Supplementary Figure 20: Key measures of interest for synaesthetes with hearing motion synaesthesia (n = 23 in the AV task and n = 31 in the VV task) and all other synaesthetes: the rate of conditioned hallucinations (AV task in **A**, VV task in **B**), estimated parameter ν (indexing the relative weight of the prior compared to the sensory evidence; AV task in **C**, VV task in **D**), β^{-1} (indexing stochasticity of response; AV task in **E**, VV task in **F**), and ω_2 (indexing the estimate of baseline environmental volatility; AV task in **G**, VV task in **H**). Error bars depict the standard error of the mean. p <0.05, ** p <0.01, *** p <0.001

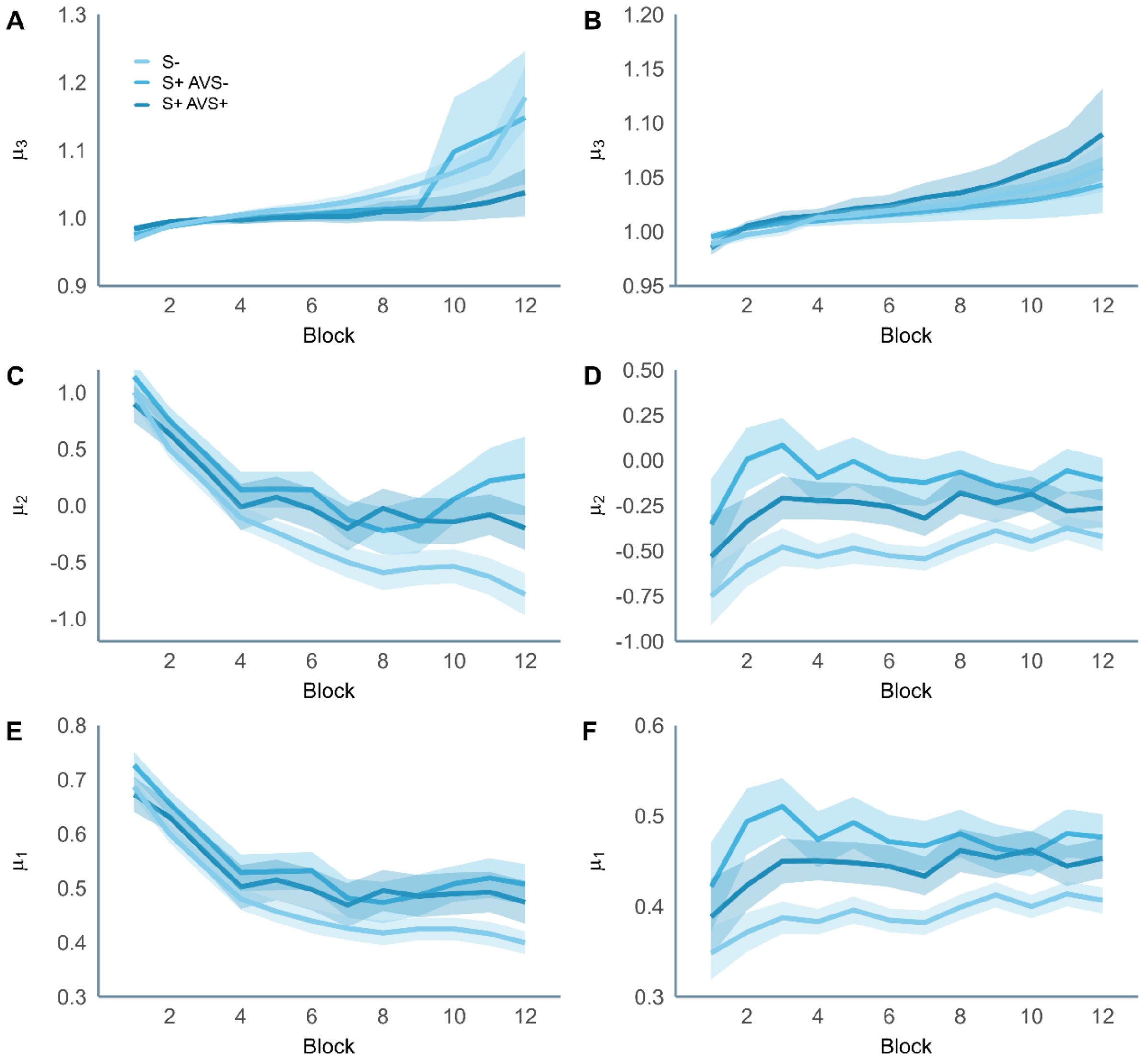

Supplementary Figure 21: Estimated belief trajectories per task for synaesthetes with auditory-to-visual synaesthesia (n = 32 in the AV task and n = 41 in the VV task), all other synaesthetes, and controls: X_1 represents the belief that the conditioned stimulus was present or not on a given trial (AV task in **E**, VV task in **F**), X_2 represents the belief that the cue and the conditioned stimulus are associated (AV task in **C**, VV task in **D**), and X_3 represents the belief in the volatility of the association (AV task in **A**, VV task in **B**) , where $\mu_{1\text{-}3}$ is the current belief or posterior at the corresponding level $X_{1\text{-}3}$. Shaded regions depict the standard error of the mean.

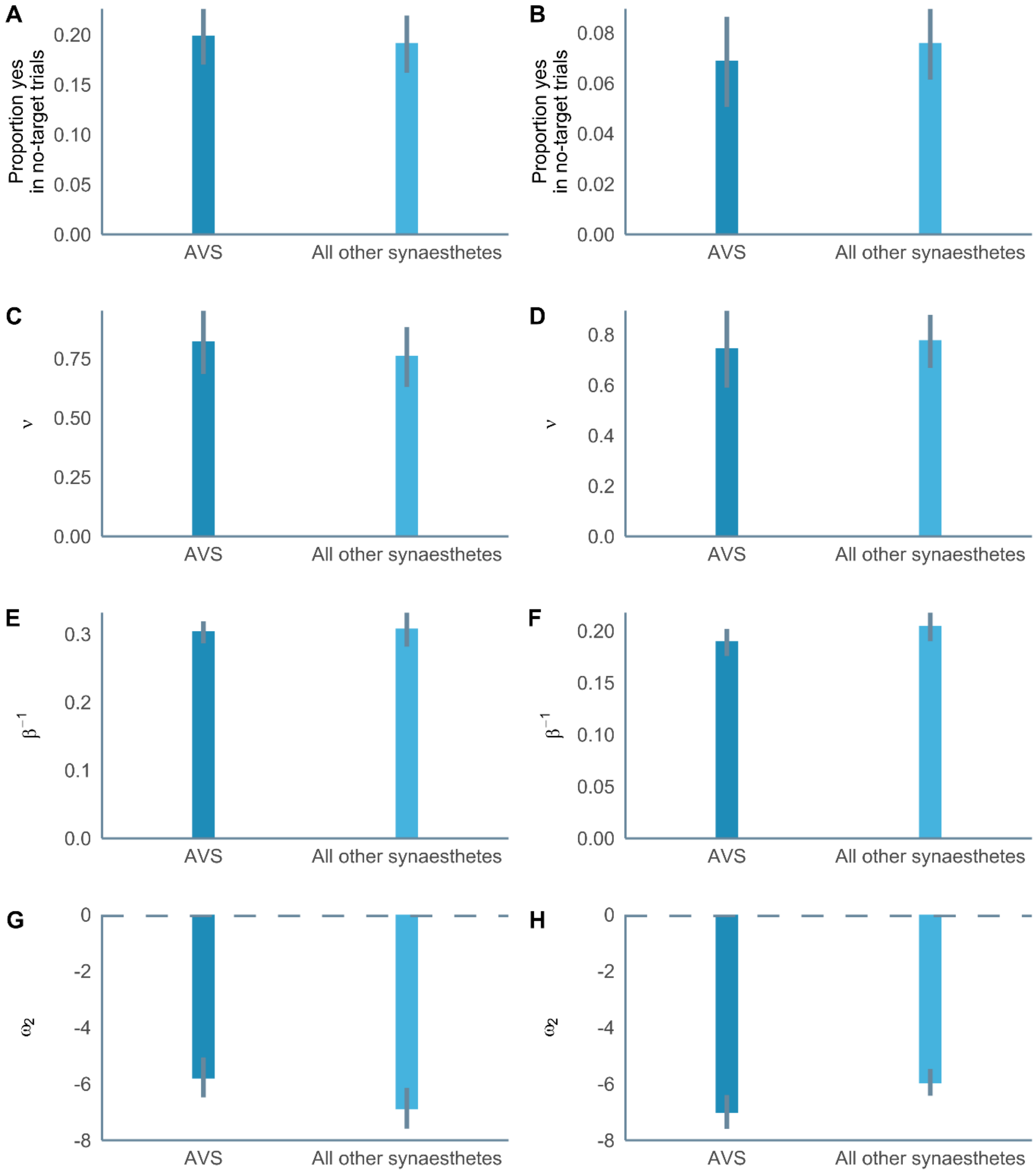

Supplementary Figure 22: Key measures of interest for synaesthetes with auditory-to-visual synaesthesia((n = 32 in the AV task and n = 41 in the VV task) and all other synaesthetes: the rate of conditioned hallucinations (AV task in **A**, VV task in **B**), estimated parameter ν (indexing the relative weight of the prior compared to the sensory evidence; AV task in **C**, VV task in **D**), β^{-1} (indexing stochasticity of response; AV task in **E**, VV task in **F**), and ω_2 (indexing the estimate of baseline environmental volatility; AV task in **G**, VV task in **H**). Error bars depict the standard error of the mean. p <0.05, ** p <0.01, *** p <0.001

8. Exploratory follow-up analysis: prediction error precision

Given the pattern in the belief trajectories reflecting diminished updating in synaesthesia, it was plausible that the prediction error was underweighted. We therefore compared the average parameters ψ and ε at level X_2 between synaesthetes and controls (see Supplementary Figure 23), as these index the precision weight on the prediction error and the precision-weighted prediction error, respectively. While ψ_2 tends to be lower in synaesthetes, consistent with the fixed belief trajectories, the difference is not significant (AV: $t(84.152) = 0.178$, $p = 0.859$; VV: $t(149.26) = 0.775$, $p = 0.440$). There are no consistent differences in ε_2 (AV: $t(134.87) = -1.081$, $p = 0.281$; VV: $t(130.3) = -1.197$, $p = 0.233$).

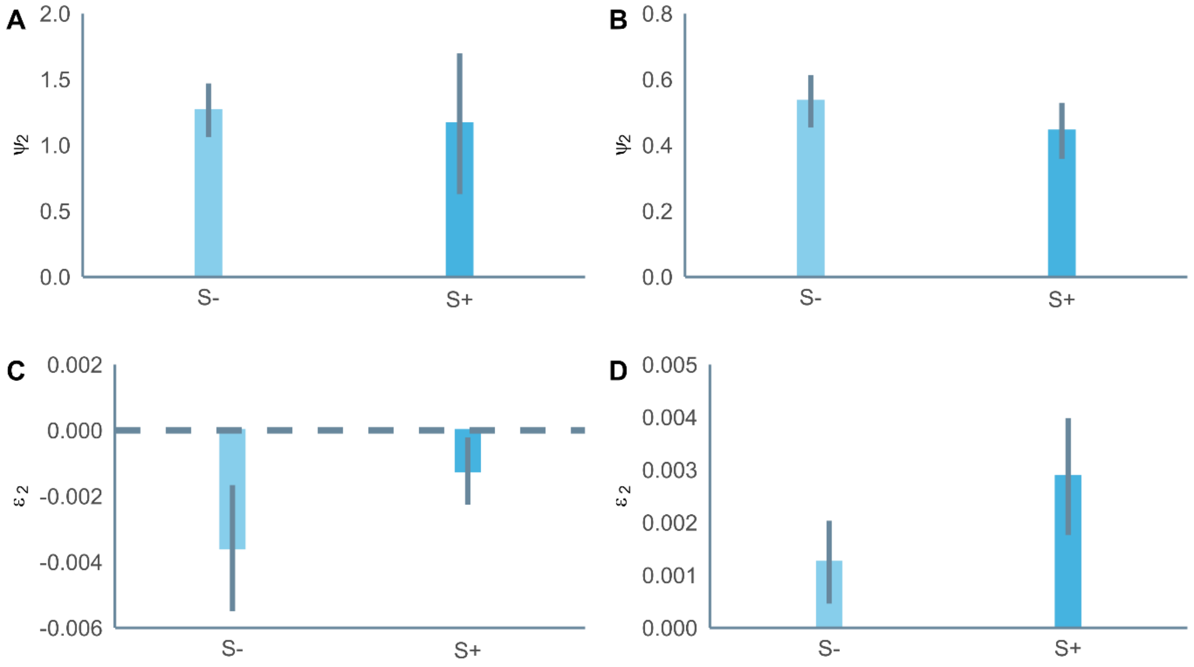

Supplementary Figure 23: Estimated average parameters ψ and ε at level X_2 for synaesthetes and controls in the AV task (**A** and **C** respectively) and the VV task (**B** and **D** respectively). Error bars depict the standard error of the mean.

9. Exploratory follow-up analysis: confidence reports

Confidence ratings provide a behavioural measure of participants' own estimate of reliability. Indeed, individuals prone to hallucinations have significantly higher confidence ratings in yes-responses in no-target trials together with a higher rate of yes responses[1,7]. Conversely, synaesthetes do not differ from controls in their confidence ratings in detection reports in no-target trials in either task (AV: $t(125.4) = -0.140$, $p = 0.889$; VV: $t(134.86) = 0.304$, $p = 0.762$), while they tend to report higher confidence in detections in target-present trials, particularly for yes responses and in the VV task (see Supplementary Figure 24). This was explored using 3-way repeated measures ANOVAs with the factors response type, condition, and group. While the trends are similar across tasks, there are no

significant effects of synaesthesia in the AV task. However, in the VV task there is a significant interaction between synaesthesia and signal intensity ($F(2.64, 353.18) = 3.440$, $p = 0.022$) as well as between synaesthesia, response type and signal intensity ($F(1.95, 261.65) = 4.090$, $p = 0.019$).

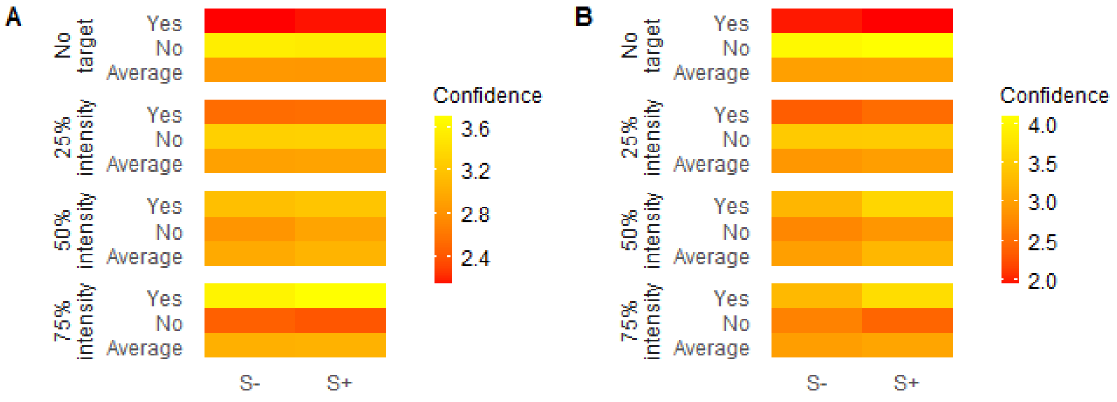

Supplementary Figure 24: Average confidence ratings for synaesthetes and controls per response type and condition in **A** the AV task and **B** the VV task.

10. References

1. Kafadar, E. *et al.* Conditioned Hallucinations and Prior Overweighting Are State-Sensitive Markers of Hallucination Susceptibility. *Biol. Psychiatry* (2022) doi:10.1016/j.biopsych.2022.05.007.
2. Woods, K. J. P., Siegel, M. H., Traer, J. & McDermott, J. H. Headphone screening to facilitate web-based auditory experiments. *Atten. Percept. Psychophys.* **79**, 2064–2072 (2017).
3. Kleiner, M. *et al.* What's new in psychtoolbox-3. *Perception* **36**, 1–16 (2007).
4. Watson, A. B. & Pelli, D. G. Quest: A Bayesian adaptive psychometric method. *Percept. Psychophys.* **33**, 113–120 (1983).
5. Ho, D., Imai, K., King, G. & Stuart, E. A. MatchIt: Nonparametric Preprocessing for Parametric Causal Inference. *J. Stat. Softw.* **42**, 1–28 (2011).
6. Stephan, K. E., Penny, W. D., Daunizeau, J., Moran, R. J. & Friston, K. J. Bayesian model selection for group studies. *NeuroImage* **46**, 1004–1017 (2009).
7. Powers, A. R., Mathys, C. & Corlett, P. R. Pavlovian conditioning–induced hallucinations result from overweighting of perceptual priors. *Science* **357**, 596–600 (2017).
